# Operando film-electrochemical EPR spectroscopy tracks radical intermediates in surface-immobilized catalysts

**DOI:** 10.1038/s41557-024-01450-y

**Published:** 2024-02-14

**Authors:** Maryam Seif-Eddine, Samuel J. Cobb, Yunfei Dang, Kaltum Abdiaziz, Mark A. Bajada, Erwin Reisner, Maxie M. Roessler

**Affiliations:** 1 Department of Chemistry, Molecular Sciences Research Hub, Imperial College London, London, UK; 2 Yusuf Hamied Department of Chemistry, University of Cambridge, Cambridge, UK; 3 Present Address: Max Planck Institute for Chemical Energy Conversion, Mülheim an der Ruhr, Germany

**Keywords:** Catalytic mechanisms, Electron transfer, Electrocatalysis, Electrocatalysis

## Abstract

The development of surface-immobilized molecular redox catalysts is an emerging research field with promising applications in sustainable chemistry. In electrocatalysis, paramagnetic species are often key intermediates in the mechanistic cycle but are inherently difficult to detect and follow by conventional in situ techniques. We report a new method, operando film-electrochemical electron paramagnetic resonance spectroscopy (FE-EPR), which enables mechanistic studies of surface-immobilized electrocatalysts. This technique enables radicals formed during redox reactions to be followed in real time under flow conditions, at room temperature and in aqueous solution. Detailed insight into surface-immobilized catalysts, as exemplified here through alcohol oxidation catalysis by a surface-immobilized nitroxide, is possible by detecting active-site paramagnetic species sensitively and quantitatively operando, thereby enabling resolution of the reaction kinetics. Our finding that the surface electron-transfer rate, which is of the same order of magnitude as the rate of catalysis (accessible from operando FE-EPR), limits catalytic efficiency has implications for the future design of better surface-immobilized catalysts.

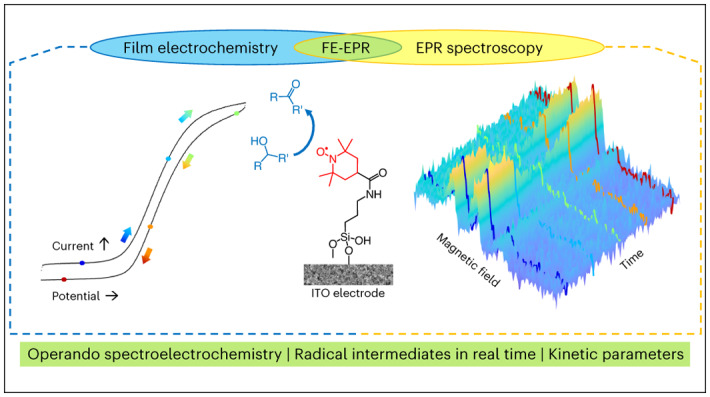

## Abstract (web summary)

Although surface-bound molecular catalysts offer well-defined active sites on heterogeneous supports, it is challenging to identify key radical intermediates in the reaction mechanism. Now, a characterization method has been developed that combines film electrochemistry and EPR spectroscopy to track radical intermediates in real time, exemplified by alcohol oxidation with a surface-immobilized nitroxide.

## Main

**Fig. 1:**
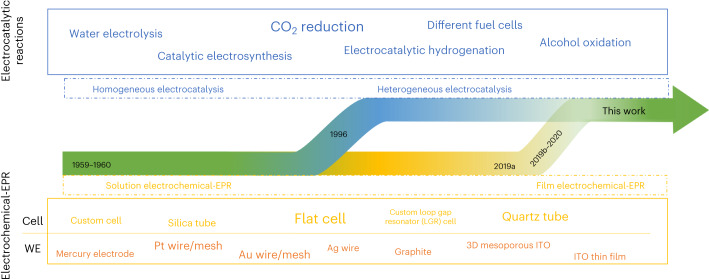
Evolution and example applications of homogeneous and heterogeneous electrocatalysis and solution or film-electrochemical EPR set-ups. The blue arrow represents the transformation from homogeneous electrocatalysis to the more sustainable heterogeneous electrocatalysis. Examples of electrocatalytic reactions given in the blue box are inspired by refs. ^10,11,72^. In nitroxide-catalysed alcohol oxidation, solution-based molecular electrocatalysts constituted the first generation, and electrode-bound film-electrocatalysts form the second generation^34^. The yellow arrow represents the transition from solution-electrochemical EPR to film-electrochemical EPR, with examples of cells and working electrodes (WEs) used throughout recent decades as listed in ref. ^3^. The text size reflects the representation of the concept or topic in the literature. The dates given refer to the following representative publications: 1959^73^, 1960^74^, 1966^75^, 2019a^76^, 2019b^19^ and 2020^36^.

Electron paramagnetic resonance (EPR) spectroscopy is used to investigate both homogeneous and heterogeneous catalysis^1–3^ and helps to guide the development of new catalysts. Although homogeneous catalysts are commonly employed in organic synthesis laboratories due to their high activity, heterogeneous catalysts are attractive as they are more easily recyclable and economic, and dominate in 80% of industrial processes^4,5^. The need for sustainable, efficient and selective chemical processes is driving the ‘heterogenization’ of homogeneous redox catalysts (Fig. 1), combining the advantages of both catalyst types to develop electrocatalysts for a future net-zero chemical industry^4–7^. Electrocatalysis has applications in fuel cells, hydrogenation, CO_2_ reduction^8–10^, organic electrosynthesis^11^ and photoelectrosynthesis^12^. Compared to traditional methods, electrocatalysis promises to be a greener alternative because it produces less metal waste, operates under mild conditions, requires shorter reaction times and is industrially scalable^13^. Innovating electrocatalysts at a time when green electricity capacity and electrification provide an appealing prospect for a net-zero economy is therefore an important aim towards a sustainable future^5,6,10,14,15^. The innovation of electrocatalysts requires knowledge of catalytic processes on surfaces and the associated challenges when catalysts are heterogenized, as understanding from catalysts in solution provides only a guide and is not directly transferable.

There is a long, but discontinuous, history of combining electrochemistry with EPR to investigate radical intermediates in catalytic reactions^3,16^ (Fig. 1). Different spectroelectrochemical (SEC) cells and working electrodes (WEs) have been proposed, and solution-electrochemical EPR has provided insight into homogeneous electrocatalysis^3^. Combining film electrochemistry with EPR is thus needed to complement the heterogenization of molecular electrocatalysts, and this requires a suitable cell and WE. The most common cells are flat-cells^3^, with EPR spectra collected using chronoamperometry in conjunction with spin traps^17^, or freeze-quenching^18,19^, to detect short-lived intermediates. However, it has not yet been possible to monitor radicals in real time under catalytically relevant conditions, with direct potential control. Given that operando EPR techniques have recently helped to understand battery processes^20,21^ and heterogeneously catalysed gas-phase reactions^22,23^, the development of operando electrochemical EPR to obtain kinetic resolution of surface-immobilized electrocatalytic reactions and direct insight into paramagnetic active-site species is timely.

A key bottleneck in the development of operando electrochemical EPR lies in the seemingly incompatible requirements of the two techniques involved: maximizing the conductivity for electrochemistry and minimizing it for EPR measurements, while also maintaining sensitivity. The technical challenges are amplified during catalysis, where mass transport limitations should be avoided, requiring flow conditions. Electrocatalysis in water (an ideal green solvent) poses a further challenge for room-temperature EPR, given its high dielectric constant, and constraints such as geometry, background signals, functionalizability and sensitivity limit the choice of WE^3^. Our ex situ film-electrochemical EPR (FE-EPR) methodology enables direct potential control of buried redox centres in proteins^19^, using hierarchical indium tin oxide (ITO) WE materials, which are highly tunable, functionalizable and common substrates for use in a wide range of electrocatalysts^24–26^. However, the capabilities of this method have been limited because it requires frozen samples (to circumvent the high dielectric constant of water) and uses chronoamperometry to hold the potential ex situ before the EPR measurements^19,27^, thus preventing visualization of active-site species and kinetic resolution of catalytic reactions, which requires an operando method.

In this Article we describe our development of an in situ FE-EPR set-up that detects active-site radical intermediates during electrocatalysis, in real time, under flow conditions and potential control. FE-EPR can selectively and very sensitively detect radical intermediates with kinetic resolution, and complements other operando spectroelectrochemical methods such as infrared^25^, Raman^25,28^ and UV–vis^29^. We use nitroxide-catalysed alcohol oxidation as a model system to demonstrate the insights that can be gained into surface-immobilized catalysts using operando FE-EPR. Nitroxides have been used extensively as electrocatalysts given their environmentally friendly and efficient conversion of alcohols to carbonyls^30–33^. Recent work has shifted towards film-electrocatalysis^27,34,35^ to improve recyclability, although this immobilization brings challenges. The capability to monitor and quantify active-site radical intermediates using operando FE-EPR provides a platform to resolve key challenges introduced by surface anchorage of the catalyst, and paves the way towards understanding and designing surface-immobilized catalysts with enhanced performance.

## Results and discussion

### Concept and demonstration of operando FE-EPR

**Fig. 2:**
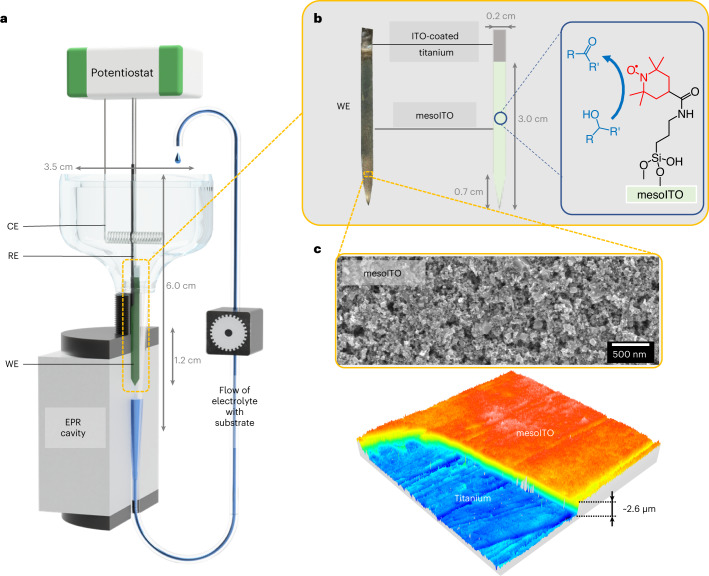
FE-EPR set-up and WE design. **a**, Cell design and assembled FE-EPR set-up with a three-electrode configuration (not drawn to scale). Ti wire connects the mesoporous indium tin oxide (mesoITO) WE to the potentiostat. RE = Ag|AgCl (3 M KCl); CE = Ni wire (for cost efficiency and greater sustainability). The *iR* drop was minimal at 27 Ω (Supplementary Fig. 6 and Supplementary Table 2). **b**, mesoITO electrode (left, photographic image; right, schematic representation). **c**, SEM surface image (top) and 3D view of the confocal topography (bottom, area = 321 × 321 μm^2^) of the mesoITO electrode (Supplementary Fig. 1). Source data

The SEC cell (Fig. 2a) was designed to collect EPR spectra synchronously with a suite of common electrochemical techniques, allowing analysis of catalytic systems. The goal was to maximize the quality factor for EPR sensitivity while achieving an electrochemical performance equivalent to a standard three-electrode electrochemical cell, even during operando conditions. The materials to assemble the SEC cell were selected based on the requirements to be EPR-silent, microwave-transparent and electrochemically inert. The cell consists of a conical lower part, which contains the WE and is inserted into the EPR cavity, and a three-dimensionally (3D)-printed upper ‘reservoir’ (see Methods for further details), which contains the counter electrode (CE). The reference electrode (RE) transcends both parts but does not enter the EPR cavity. The conical shape keeps the RE and WE in close proximity and enables effective electrolyte mass transport between electrodes. The inclusion of a RE (as opposed to a pseudo-RE^36,37^) ensures accurate potential readings. In contrast to our set-up, which also enables cyclic voltammetry (CV), previous electrochemical EPR measurements applied chronoamperometry, followed by acquisition of EPR spectra^36,38,39^.

To maximize the electrochemical and EPR sensitivity, the surface area of the WE was augmented by assembling a 2.6 µm layer of mesoITO (self-assembled from ITO nanoparticles; Fig. 2c and Supplementary Fig. 1) onto both sides of an ITO-coated (10 nm) flat titanium strip with a total geometric surface area of ~100 mm^2^ (Fig. 2b). ITO was chosen because of its highly tunable topology compared to other electrodes and its ability to be assembled into hierarchical structures with different pore sizes^26^. It is compatible with a wide range of redox systems, including small-molecular catalysts^27,40,41^, enzymes^24,42^, whole cells^43^ and bacteria^44^. This electrode exhibited a much faster current response (0.3 s; Supplementary Fig. 2) than our previous ITO electrodes for SEC (~300 s)^19,27^ and other electrochemical EPR set-ups^39,45^, a prerequisite for CV-based SEC. TEMPO (2,2,6,6-tetramethyl-1-piperidinyloxyl) with a silatrane anchoring group (STEMPO) was immobilized onto the mesoITO electrodes^27,46^ (STEMPO|mesoITO; Fig. 2b) and shown to be surface-bound (Supplementary Fig. 4).

**Extended Data Fig. 1:**
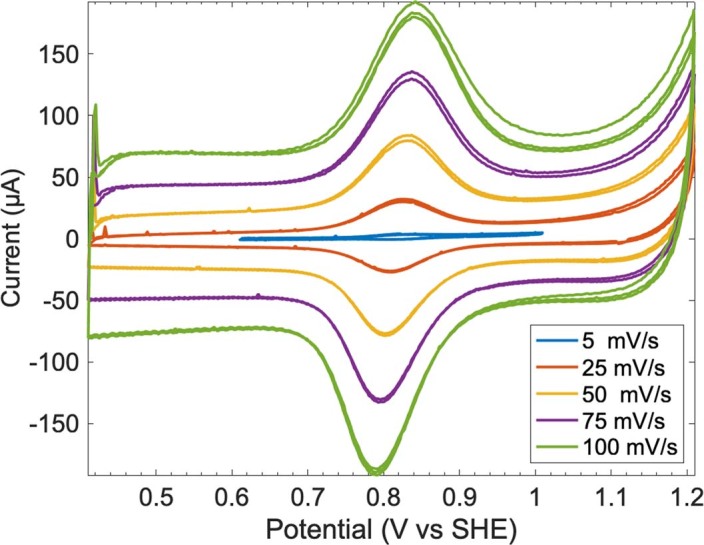
Electrochemical characterisation of STEMPO|mesoITO electrode in the FE-EPR cell. Cyclic voltammograms of STEMPO|mesoITO electrode showing oxidoreduction of the STEMPO^•^/STEMPO^+^ redox couple in the FE-EPR cell with 8 ml 500 mM Na_2_CO_3_(aq), pH 8.0 at 20 °C. Two CVs are shown for each scan rate to illustrate the stability. See methods for experimental parameters and conditions. Source data

**Extended Data Fig. 2:**
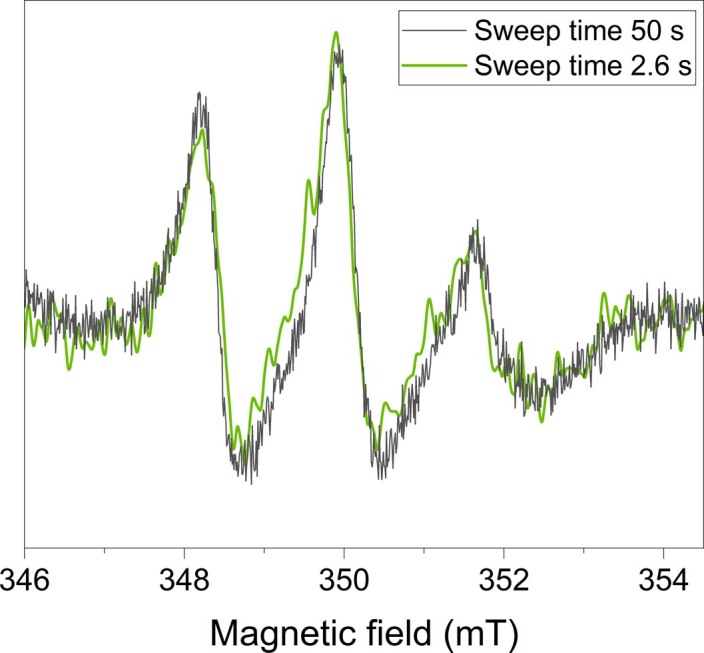
EPR characterisation of STEMPO|mesoITO electrode in the FE-EPR cell. Long scan (black line, 50 s) and short scan (green line, 2.6 s) EPR spectra of STEMPO^•^ attached to the surface of ITO measured in the FE-EPR cell at open circuit potential in presence of buffer and under flow conditions. See Methods for experimental parameters and conditions. Source data

The FE-EPR set-up was evaluated both electrochemically and coupled to EPR. CV scans of STEMPO|mesoITO in the FE-EPR cell at different scan rates exhibit the same electrochemical response as those in a standard electrochemical cell and were unchanged under anaerobic conditions (Extended Data Fig. 1 and Supplementary Section 2). EPR spectra of STEMPO|mesoITO inserted inside the FE-EPR cell in the presence of aqueous buffer solution showed a typical nitroxide signal in the slow-motion regime, consistent with a surface-bound radical (Extended Data Fig. 2). To maximize the signal-to-noise ratio, experiments were performed with the highest non-saturating microwave power and a high (0.4 mT) modulation amplitude. Short sweep times (2.6 s) minimized the potential error of the continuously acquired EPR spectra in the FE-EPR experiments (Supplementary Section 3). Although this allowed the acquisition of 75 EPR spectra per CV scan spanning 800 mV at 5 mV s^−1^, fast scan rates could face a limitation arising from the time required for a single EPR field sweep (here 2.6 s, resulting in a 21 mV ‘window’ per EPR spectrum).

### FE-EPR of STEMPO under non-turnover conditions

**Fig. 3:**
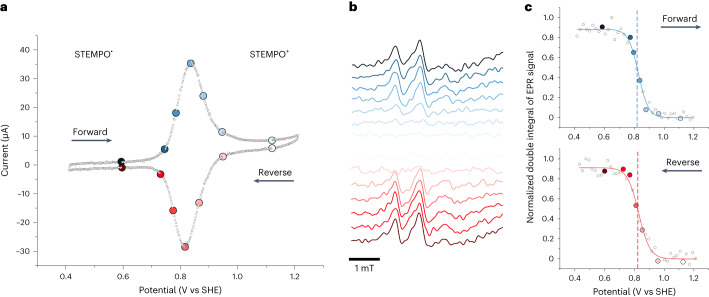
Oxidoreduction of STEMPO^•^/STEMPO^+^, monitored with in situ FE-EPR. **a**, CV scan recorded at 5 mV s^−1^, simultaneously with EPR measurements, in the FE-EPR cell placed inside the EPR cavity. The scan direction is indicated with arrows. Peak separation, 19 mV, *E*_(non-catalytic, CV)_ = +830 mV. **b**, EPR spectra at the potentials highlighted on the CV scan in **a** during the forward (blue) and reverse (red) CV scans (Extended Data Fig. 3 presents a complete set of EPR spectra). **c**, Double integrals of the EPR spectra (normalised to the maximum data point) plotted against potential and fitted with a 1e^−^ Nernst equation during forward (blue, *E*_forward(non-catalytic, FE-EPR)_ = +831 mV) and reverse (red, *E*_reverse(non-catalytic, FE-EPR)_ = +830 mV) CV scans. Grey open circles are from all EPR spectra collected (Extended Data Fig. 3), and coloured circles correspond to the EPR data shown in **b**. Source data

**Extended Data Fig. 3:**
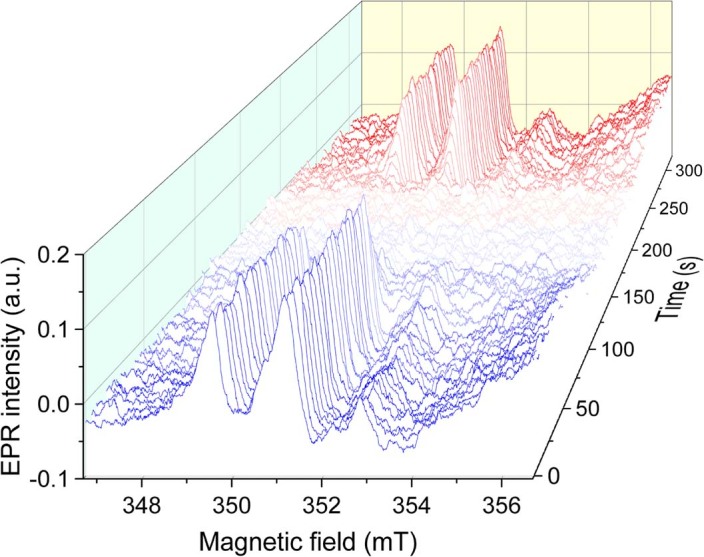
Complete set of EPR spectra recorded over the course of the FEcv-EPR experiment under non-catalytic conditions. Complete set of 77 EPR spectra obtained during the FE_CV_-EPR measurements during both forward and reverse CV scans. The sweep time per spectrum was 2.6 s, and around 4 s including processing time. With a scan rate of 5 mVs^−1^ and a potential range of 800 mV, each EPR spectrum has a potential range of 21 mV. During the forward CV scan, the intensity of the EPR spectra decreases with increasing the potential, reaching a minimum at 900 mV. During the reverse CV scan, the intensity increases back to reach a maximum at 800 mV. Source data

**Extended Data Fig. 4:**
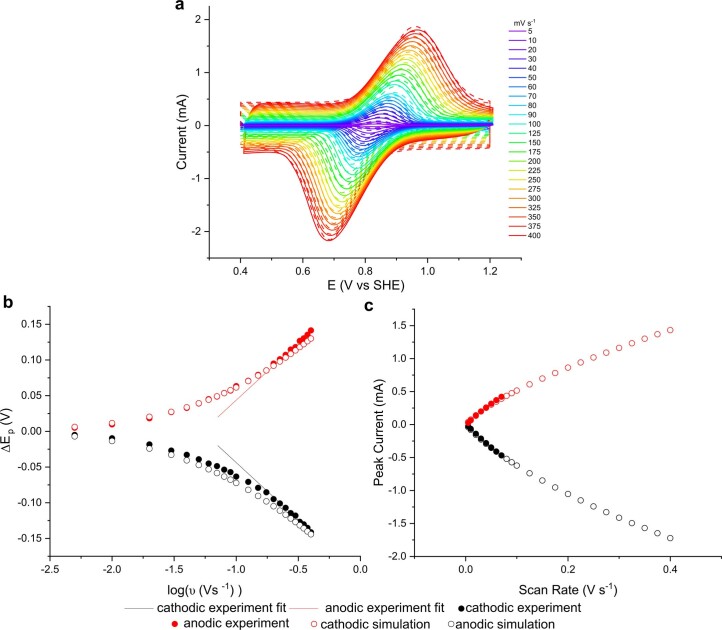
Experimental and simulated non-turnover signals for STEMPO. **a** Experimental (dashed) and simulated (solid) cyclic voltammograms of STEMPO on a MesoITO electrode over a range of scan rates (5–400 mV) at pH 8.0. For Experimental details see Methods and for simulation details see Supplementary Information section 9. **b** Experimental (filled points) and simulated peak potentials for the anodic (red) and cathodic (black) branch. Lines represent linear fits to the region of experimental data where Δ*E*_p_ > 200 mV for experimental determination of *k*_s_ = 0.77 s^−1^ and *α* = 0.37. **c** Experimental (filled points) and simulated peak currents for the anodic (red) and cathodic (black) peak potentials as a function of scan rate. Source data

The surface-immobilized STEMPO^•^/STEMPO^+^ redox reaction was investigated using FE-EPR. The EPR spectra were recorded continuously while sweeping the potential. Figure 3a and Supplementary Video 1 present an example CV scan measured at 5 mV s^−1^, with a selected set of corresponding EPR spectra shown in Fig. 3b (the complete set is shown in Extended Data Fig. 3). Double integration of the STEMPO radical EPR spectra from the forward and reverse scans, plotted as a function of potential (Fig. 3c), allow deduction of the reduction potential of the reversible STEMPO^•^/STEMPO^+^ redox couple (*E*_(non-catalytic, FE-EPR)_ = +830 mV) by fitting the data to the Nernst equation with the number of electrons transferred (*n*) equal to 1. The surface electron-transfer rate constant (*k*_s_) was determined from Laviron analysis (*k*_s_ = 0.77 s^−1^; Supplementary Fig. 4) and agrees with previous work^27^. The potential derived from EPR measurements was in close agreement with that obtained from the CV scan (Fig. 3 and Supplementary Table 1), demonstrating the excellent performance of the FE-EPR in situ set-up. Multiphysics simulations (COMSOL Multiphysics; Supplementary Section 9) of the FE-EPR data in Fig. 3 enabled us to build a more complete model of electron transfer based on asymmetric Marcus–Hush–Chidsey theory, incorporating intermolecular interactions^47,48^ (Extended Data Fig. 4), which is fully consistent with the experimental data obtained from FE-EPR experiments across the entire range of scan rates used.

### FE-EPR of STEMPO under catalysis

**Fig. 4:**
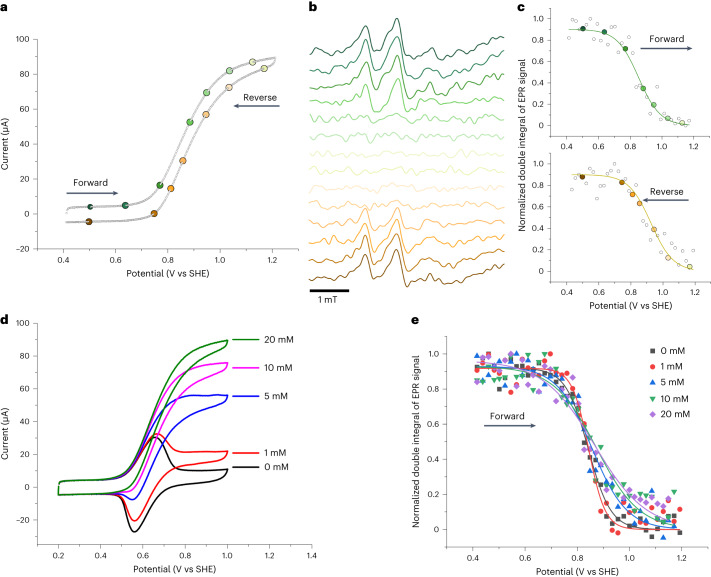
Alcohol catalysis by surface-immobilized nitroxide (STEMPO) monitored with operando FE-EPR. **a**, CV scan from the FE-EPR cell placed inside the EPR cavity, recorded at 5 mV s^−1^ with 20 mM MBA in 8 ml of buffer solution, pH 8.0, in air and under flow conditions. **b**, EPR spectra at the potentials highlighted on the CV scan in **a** during forward (green) and reverse (yellow) CV scans (Extended Data Fig. 6 provides a complete set of EPR spectra). **c**, Double integrals of the EPR spectra (normalised to the maximum data point) plotted against potential and fitted with the Nernst equation (apparent number of electrons transferred, *n*_app_ = 0.3) during the forward (green, *E*_forward (catalytic, FE-EPR)_ = +859 mV) and reverse (yellow, *E*_reverse(catalytic, FE-EPR)_ = +902 mV) CV scans. Grey open circles are from all EPR spectra collected (Extended Data Fig. 6), and the coloured circles correspond to the EPR data shown in **b**. **d**, Catalytic CV scans from operando FE_CV_-EPR experiments with different concentrations of MBA. **e**, Double integrals of the EPR spectra from **c**, plotted against potential and fitted with the Nernst equation during the forward CV scan (Supplementary Section 5). Source data

**Extended Data Fig. 5:**
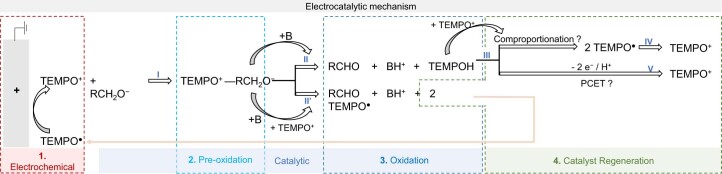
Schematic representation of the mechanism of solution-based electrocatalysis with TEMPO^•^. Electrochemical oxidation (step 1), adduct formation (step 2), alcohol oxidation (step 3) and catalyst regeneration (step 4).

Having successfully established in situ FE-EPR under non-catalytic conditions, we proceeded to investigate STEMPO as an ideal model catalyst, given that nitroxide-catalysed alcohol oxidation has been investigated in detail in solution^34^ (Extended Data Fig. 5 and Supplementary Section 4) and less extensively in the surface-immobilized form^27,35,49–51^. The surface-immobilized reaction with the substrate 4-methylbenzyl alcohol (MBA) proceeds via a catalytic electrochemical–chemical mechanism (EC′) (based on a catalytic CV scan, Fig. 4a), consistent with previous studies carried out both in solution and when surface-immobilized^34,52–54^. Interestingly, it has been shown using in situ Raman and vibrational spectroscopy that the mechanism of a Co catalyst is different on a surface^25^ than in solution^55^, highlighting that not all aspects of solution-based alcohol oxidation by nitroxides will necessarily translate to the surface-immobilized case.

**Extended Data Fig. 6:**
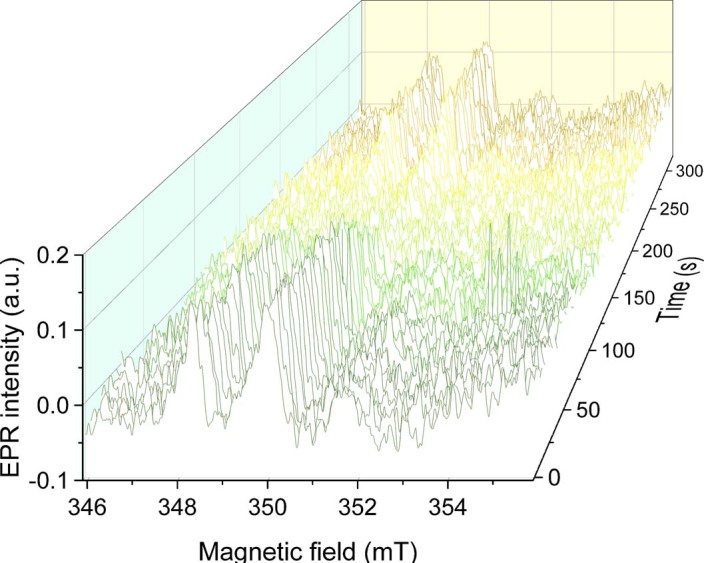
Complete set of EPR spectra recorded over the course of the FEcv-EPR experiment under catalytic conditions. EPR spectra obtained during the FE_CV_-EPR measurements during both forward and reverse CV scans in the presence of 20 mM MBA under catalytic conditions. Similarly, to the non-turnover spectra, each EPR spectrum covers ~21 mV. During the forward CV scan, the intensity of the EPR spectra decreases with increasing the potential, reaching a minimum at 1000 mV. During the reverse CV scan, the intensity increases back to reach a maximum at 700 mV. Source data

The simultaneously recorded EPR spectra (Fig. 4b; Extended Data Fig. 6 provides a complete set) show the gradual disappearance of STEMPO^•^ as it is transformed to STEMPO^+^ in the forward scan, then its reappearance in the reverse scan. The flow conditions (Fig. 2a) ensure that mass transport of the MBA is sufficiently high not to be rate-limiting, with minimal substrate depletion at the electrode surface (Supplementary Fig. 26). The same EPR spectrum was regenerated in the reverse scan, suggesting chemical reversibility by reforming a radical species, after catalysis, that is structurally the same as the one present before catalysis, and providing evidence that STEMPO^•^ is a catalytic intermediate. In solution-based studies, TEMPO^•^ has not been detected directly during catalysis due to a lack of suitable techniques, although a radical has been inferred^56^; this showcases the sensitivity and time resolution possible with FE-EPR. Although FE-EPR is blind to diamagnetic intermediates, in this case STEMPOH and STEMPO^+^, it has the advantage of being able to selectively detect very low concentrations of key paramagnetic surface-immobilized active-site species (TEMPO^•^) that are very difficult to access with other SEC methods. In surface-immobilized electrocatalysis, this selectivity is particularly advantageous, given the size and complexity of the system.

Fitting of the double-integral EPR data in Fig. 4c with the Nernst equation provides directly observable metrics from FE-EPR to describe the catalytic system. Under catalysis, the apparent *n* (*n*_app_) decreases successively with increased substrate concentration (*n*_app_ = 0.3 for 20 mM MBA; Supplementary Table 2) in both the forward and reverse scans. Concomitantly, the catalytic potential (*E*_cat_) becomes more positive with increasing substrate concentration (Fig. 4e and Supplementary Table 2). Similar trends were observed under anaerobic conditions and with the secondary alcohol glycerol as substrate (Supplementary Section 5). *E*_catalytic_ from FE-EPR (*E*_catalytic, FE-EPR_) and the electrochemistry alone (*E*_catalytic, CV_) are in near-perfect agreement (Supplementary Table 2) in this case, where ideal sigmoidal catalytic CV scans^57^ are observed. However, it is noteworthy that FE-EPR provides this useful parameter even when electrochemical data alone cannot.

A set of rate equations was assembled for the possible processes that are taking place (equations (1) to (6)). These form the basis of kinetic investigations where the rates of some components of this scheme can be directly accessed by FE-EPR and used in combination with Multiphysics simulations for mechanistic determinations (see Methods and Supplementary Information section 9):

1 $${\rm{STEMPO}}^{{\bullet }}\,\mathop{\longrightarrow}\limits^{{{R}}_{\rm{et}}}\,{\rm{STEMPO}}^{+}+{\rm{e}}^{-}$$

2 $${\rm{STEMPO}}^{+}+{\rm{R}}-{\rm{OH}}\mathop{\rightleftharpoons }\limits^{{{K}}_{\rm{eq},\,1}}{\rm{STEMPO}}^{+}-{\rm{adduct}}$$

3 $${\rm{STEMPO}}^{+}+{\rm{OH}}^{-}\mathop{\rightleftharpoons }\limits^{{K}_{{\rm{eq}},\,2}}{\rm{STEMPO}}-{\rm{OH}}$$

4 $${\rm{STEMPO}}^{+}-{\rm{adduct}}+{\rm{OH}}^{-}\mathop{\longrightarrow }\limits^{{k}_{\rm{cat}}}{\rm{STEMPOH}}+{\rm{R}}-{\rm{CHO}}$$

5 $${\rm{STEMPOH}}+{\rm{STEMPO}}^{+}\mathop{\longrightarrow }\limits^{{k}_{\rm{comp}}}2{\rm{STEMPO}}^{{\bullet }}$$

6 $${\rm{STEMPOH}}+{\rm{OH}}^{-}\mathop{\longrightarrow }\limits^{{{R}}_{\rm{PCET}}}{\rm{STEMPO}}^{{\bullet }}+{\rm{e}}^{-}+{\rm{H}}_{2}{\rm{O}}$$

**Extended Data Fig. 7:**
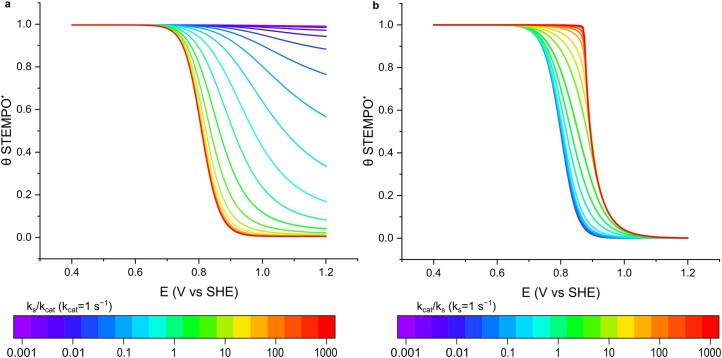
Effect of varying *k*_S_ and *k*_cat_ on the catalytic response. CVs with varied ratios of *k*_*s*_ to *k*_*cat*._ **a**, STEMPO^•^ partial surface coverages, demonstrating a STEMPO^•^ concentration dependence that fits an apparent *n* = 1 Nernst curve at high *k*_s_, decreasing as *k*_s_ < critical scan rate. **b**, STEMPO^•^ partial surface coverages, demonstrating a STEMPO^•^ concentration dependence that fits an apparent *n* = 1 Nernst curve at the limits of *k*_cat_ and an *n* < 1 apparent Nernst curve at intermediate values. The apparent *E*_1/2_ shifts to more positive potentials with increasing *k*_cat_. At high *k*_cat_ the reverse response differs from the forward response due to substrate depletion. Source data

The observed trend in *n*_app_ and *E*_catalytic_ can be explained as arising from relatively slow electron transfer, occurring at a comparable rate to catalysis (Extended Data Fig. 7 and Supplementary Section 9). When the rate constant for chemical catalysis (*k*_cat_) was fixed and *k*_s_ was decreased, *n*_app_ from the modelled STEMPO^•^ concentrations decreased. At high *k*_s_ (greater than the critical scan rate and higher than *k*_cat_), varying *k*_s_ had little effect on the wave shape. This behaviour is indicative of the interplay between *k*_s_ and *k*_cat_ in the STEMPO system, which must be of similar magnitudes (Extended Data Fig. 7), unlike in diffusion-controlled systems due to the surface-immobilized nature of the electron transfer. Although, in contrast to their homogeneous counterparts, immobilization removes diffusional components, a high *k*_s_ is essential for the performance of surface-immobilized catalysts and necessitates kinetic and mechanistic consideration. Thus, to deconvolute *k*_s_ and *k*_cat_, a direct experimental measurement of *k*_cat_ is required, highlighting the need to observe surface-immobilized catalysts directly.

### Operando FE-EPR provides kinetic resolution

**Fig. 5:**
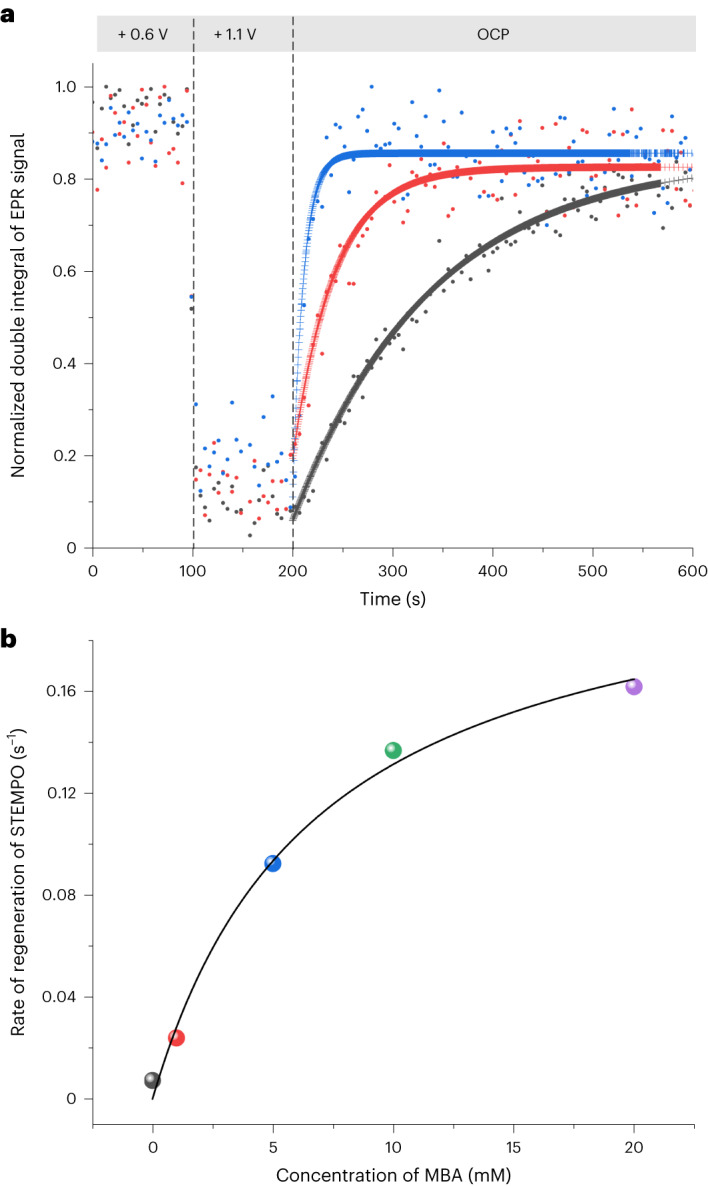
Generation of surface-immobilized EPR-active nitroxide radical intermediate (STEMPO^•^) under OCP in the absence and presence of MBA. The potential was held at +0.6 V such that only the EPR-active species (STEMPO^•^) was present, and then stepped to +1.1 V such that EPR-silent (STEMPO^+^) was generated exclusively. The current was monitored at OCP over time and the experiment was performed with and without substrate. EPR spectra were recorded throughout the chronoamperometry-OCP measurement. **a**, Double integrals of EPR spectra showing regeneration of STEMPO^•^ in the absence of substrate (black dots) and in the presence of 1 mM (red dots) and 5 mM (blue dots) MBA after potential steps at +0.6 V and +1.1 V versus SHE, for 100 s each. **b**, STEMPO^•^ regeneration rates as a function of substrate concentration (14, 92, 137 and 162 µM s^−1^ for 1, 5, 10 and 20 mM MBA, respectively; the colours of the data points match the data in **a** and in Supplementary Figure 14), deduced from a first-order relaxation fit of the variation of STEMPO^•^ concentration over time, as quantified from the EPR data. The solid line shows a fit using the Michaelis–Menten model with Michaelis–Menten constant *K*_M_ = 6.8 ± 1.3 and rate constant for chemical catalysis *k*_cat_ = 0.22 s^−1^ (see main text). Extended Data Fig. 8 provides results with glycerol as the substrate at pH 8.0 and Extended Data Fig. 9 the corresponding data for MBA at pH 7.3 (also Supplementary Section 6). Source data

**Extended Data Fig. 8:**
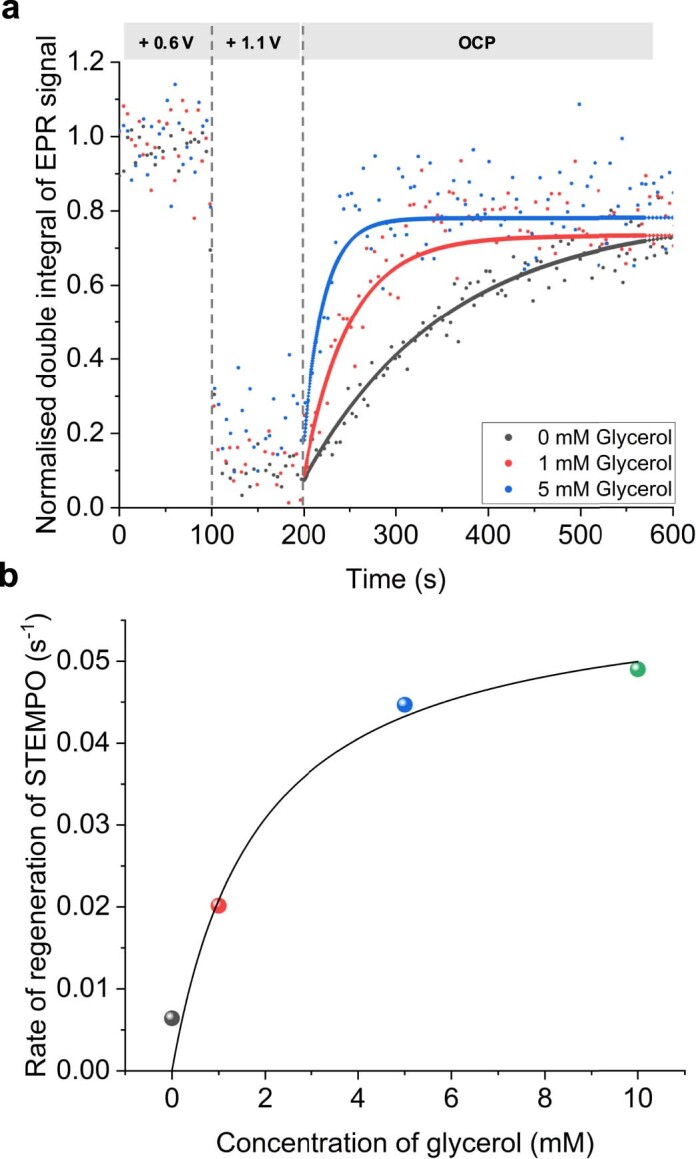
Generation of EPR-active STEMPO^•^ under open circuit potential (OCP) in the absence and presence of glycerol at pH 8.0. The potential was held at +0.6 V such that only the EPR-active species (STEMPO^•^) is present, and then stepped to +1.1 V such that EPR-silent (STEMPO + ) is generated exclusively. The current is monitored at OCP over time and the experiment was performed with and without substrate. EPR spectra were recorded throughout the chronoamperometry-OCP measurement. **a**, Double integrals of EPR spectra showing regeneration of STEMPO^•^ in the absence of substrate (black dots), and in the presence of 1 mM (red dots) and 5 mM (blue dots) glycerol after potential steps at + 0.6 V and + 1.1 V vs SHE, for 100 s each. **b**, STEMPO^•^ regeneration rates as a function of substrate concentration (0, 1, 5 and 10 mM glycerol), deduced from a 1^st^ order relaxation fit of the variation of STEMPO^•^ concentration over time, as quantified from the EPR data. The solid line shows the fit using the Michaelis-Menten model with *K*_M_ = 1.8 ± 0.9 mM and *k*_cat_ = 0.06 s^−1^. Source data

With chronoamperometry-based FE-EPR (FE_amp_-EPR), *k*_cat_ can be determined directly from operando measurements (Fig. 5), avoiding the assumptions of foot-of-the-wave analysis (Supplementary Section 10). The STEMPO^•^ EPR signal is monitored at open-circuit potential (OCP) following a high-potential step to generate STEMPO^+^ exclusively. Online product detection showed that MBA was selectively oxidized to 4-methylbenzaldehyde (Faradaic efficiency of 96%, from HPLC; Supplementary Section 8). In the absence of substrate, the STEMPO^•^ signal intensity returned to its initial levels, with a radical regeneration rate of 7.3 µM s^−1^ (Fig. 5a). Regeneration of STEMPO^•^ was also observed under anaerobic conditions at the same rate at pH 8.0 and 7.3. STEMPO^•^ regeneration was faster in the presence of MBA and increased with substrate concentration, including under anaerobic conditions. A similar trend was observed with glycerol (Extended Data Fig. 8 and Supplementary Section 6).

Regeneration of the nitroxide at OCP under catalytic conditions provides direct evidence for the formation of STEMPO^•^ as an active-site species during catalysis. The rate of STEMPO^•^ regeneration as a function of MBA or glycerol concentration fits the Michaelis–Menten model (Fig. 5b and Extended Data Fig. 8b). The Michaelis–Menten model, which is a specific case of the more general substrate saturation model (Supplementary Section 7), is based on the formation of an enzyme–substrate (catalyst–substrate) complex^58^, with binding affinity equal to the inverse of the Michaelis–Menten constant, *K*_M_. The Michaelis–Menten model, which is extensively used to characterize enzyme–substrate interactions, has rarely been applied to molecular catalysts^59–61^, but parallels can also be drawn with the Langmuir–Hinshelwood mechanism describing the surface saturation of heterogeneous catalysis^62^. Quantification of the catalyst–substrate association kinetics is then straightforward. The *K*_M_ values determined from FE-EPR for STEMPO^+^–MBA and STEMPO^+^–glycerol associations are 6.8 ± 1.3 and 1.8 ± 0.9 mM, respectively. The higher binding affinity for glycerol is expected given its three -OH groups (compared to one in MBA). The catalytic rates, *k*_cat_, are 0.22 s^−1^ for MBA and 0.06 s^−1^ for glycerol at pH 8.0, as quantified from EPR (Supplementary Fig. 10 and Supplementary Table 3). Although oxoammonium-alcohol adduct formation has been studied extensively in solution^31,34,63–66^, the substrate binding affinity is rarely quantified^67^.

**Extended Data Fig. 9:**
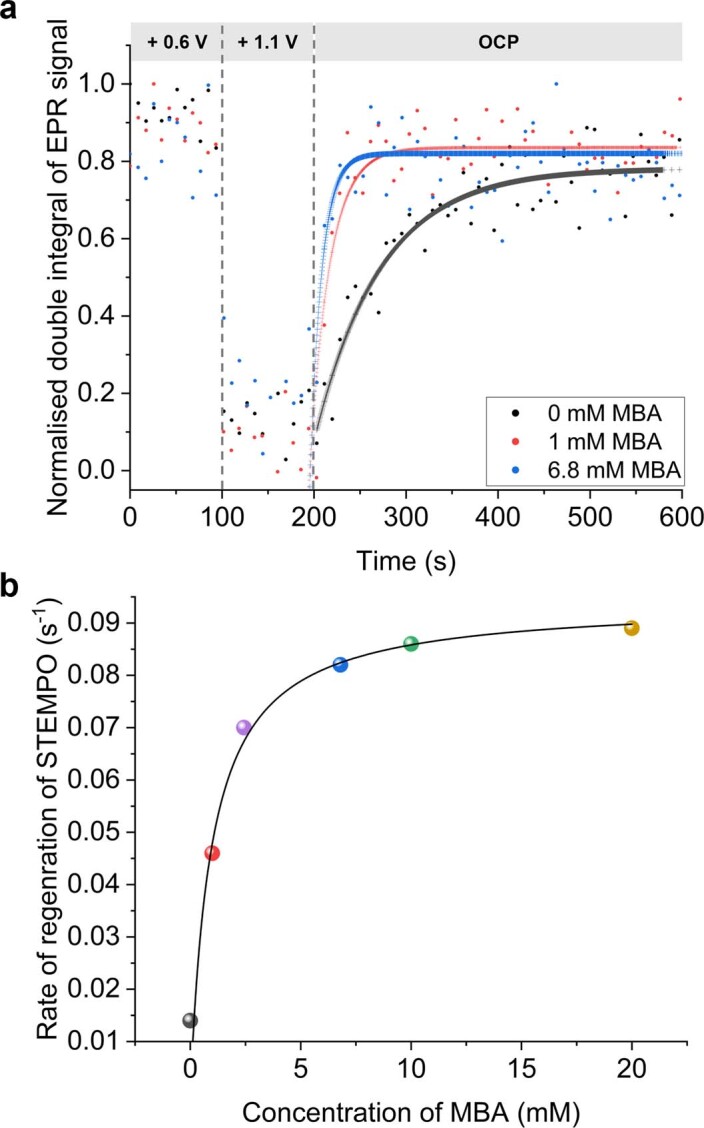
Generation of EPR-active STEMPO^•^ under open circuit potential (OCP) in the absence and presence of MBA at pH 7.3. The potential was held at +0.6 V such that only the EPR-active species (STEMPO•) is present, and then stepped to +1.1 V such that EPR-silent (STEMPO + ) is generated exclusively. The current is monitored at OCP over time and the experiment was performed with and without substrate. EPR spectra were recorded throughout the chronoamperometry-OCP measurement. **a** Double integrals of EPR spectra showing regeneration of STEMPO^•^ in the absence of substrate (black dots), and in the presence of 1 mM (red dots) and 6.8 mM (blue dots) MBA after potential steps at + 0.6 V and + 1.1 V vs SHE, for 100 s each. **b** STEMPO^•^ regeneration rates as a function of substrate concentration (0, 1, 2.4, 6.8, 10 and 20 mM MBA; the colours of the data points match the data in **a** and in Supplementary Figure 16), deduced from a 1^st^ order relaxation fit of the variation of STEMPO^•^ concentration over time, as quantified from the EPR data. The solid line shows the fit using the Michaelis-Menten model with *K*_M_ = 0.96 mM and *k*_cat_ = 0.09 s^−1^. Source data

**Extended Data Fig. 10:**
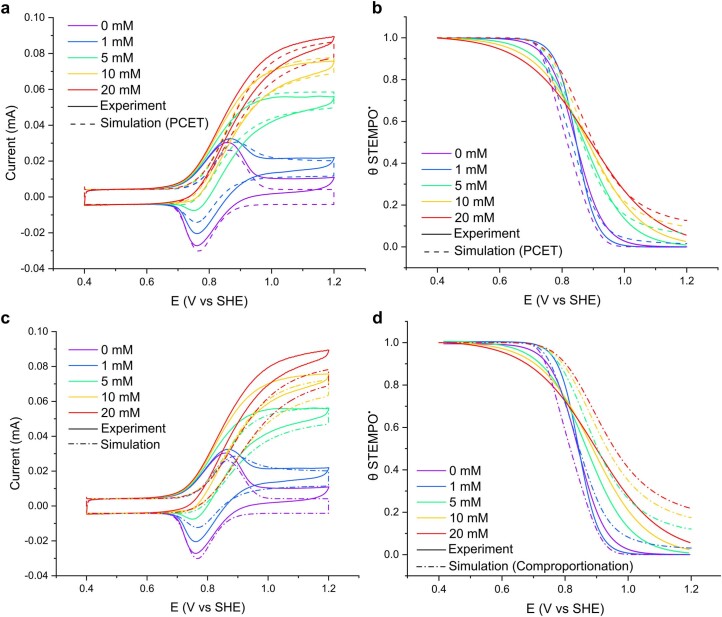
Experimental and simulated FE-EPR experiments for different STEMPO^•^ regeneration pathways. **a**, CVs at a range of substrate concentrations ([MBA] = 0–20 mM, pH 8.0) solid lines are experimental data and dash-dot lines simulate a PCET regeneration pathway **b**, STEMPO^•^ partial surface coverages at a range of substrate concentrations for a PCET regeneration pathway ([MBA] = 0–20 mM, pH 8.0) **c**, CVs at a range of substrate concentrations ([MBA] = 0–20 mM, pH 8.0) solid lines are experimental data and dash-dot lines simulate a comproportionation regneration pathway **d**, STEMPO^•^ partial surface coverages at a range of substrate concentrations for a comproportionation pathway([MBA] = 0–20 mM, pH 8.0). For all panels solid lines are fits of experimental data and dash lines are simulated data. Source data

*k*_cat_ is pH-dependent and can be directly observed by FE-EPR, with higher pH increasing *k*_cat_ and lower pH decreasing *k*_cat_ (0.09 s^−1^ at pH 7.3; Extended Data Fig. 9). The pH dependence, which has been extensively studied for different nitroxides in solution under non-catalytic conditions^68^, alludes to the formation of an oxoammonium hydroxide adduct^54^, as the rate equation dictates a linear increase with [OH^−^] (equation (4)), and a less than logarithmic dependence of the directly measured *k*_cat_ on pH is observed. This indicates a p*K*_a_ for the formation of the oxoammonium hydroxide adduct of 7.1 (equation (3)), substantially lower than that for solution TEMPO (p*K*_a_ > 12)^69^. This can be due to a structural effect from the presence of the silatrane linker^54^ or due to the effect of surface immobilization on p*K*_a_ values^25,70^. The formation of the oxoammonium-hydroxide adduct is consistent with the previously observed plateau in catalytic activity at pH 10 (ref. ^27^) and can be used to accurately describe the FE-EPR data at OCP for all substrate concentrations and pH with Multiphysics modelling (Supplementary Section 9). STEMPO^•^ is regenerated at OCP, providing evidence of a surface comproportionation reaction (Extended Data Fig. 10 and equation (5)), which would require current to flow. This is consistent with the presence of intermolecular interactions required to describe the non-turnover case. However, comproportionation does not exclude STEMPO^•^ regeneration by Proton coupled electron transfer (PCET), as the non-turnover signal for the STEMPOH/STEMPO^•^ redox couple that has been observed (with *k*_s_ = 5.7 s^−1^) is pH-dependent (*E*_0_ = 0.386 − 0.059 × pH V versus standard hydrogen electrode (SHE); Supplementary Fig. 23). It is important to emphasize that the rate-determining step is determined by *k*_cat_ (equation (4)), with comproportionation or PCET steps only influencing the regeneration of STEMPO^•^ and its concentration in the catalytic cycle.

With the availability of a measure for *k*_cat_, our electrochemical model can fully explain all trends in the extant FE-EPR data with comparable current magnitudes and wave shapes, coupled to comparable trends with substrate concentration in the EPR-observed STEMPO^•^ concentration (Extended Data Fig. 10). The direct measure of *k*_s_, *k*_cat_, the order of reactions (as deduced from *k*_cat_ at pH 8.0 versus pH 7.3) and catalytic site concentrations can be used to fix the rates of some reactions in the catalytic cycle (Supplementary Tables 3 and 6). Different mechanisms for components of the cycle that are not yet accessible directly from experiments (namely possible reactions following the rate-determining step) were then tested with our Multiphysics model. The mechanism of STEMPO^•^ regeneration after the catalytic intermediate is debated in solution-based reactions, with both comproportionation^71^ and PCET constituting possible pathways^34^ (Extended Data Fig. 10 and Supplementary Section 9), and insight into the surface-immobilized process is lacking. However, unlike in solution-phase catalysis, there is no requirement for STEMPOH to diffuse to the electrode to enable a PCET pathway. Using the experimentally determined *k*_s_ and *k*_cat_, the observed STEMPO^•^ concentrations under catalysis are more consistent with PCET being the dominant regeneration mechanism (Extended Data Fig. 10). Although this does not preclude a comproportionation mechanism alongside PCET, the PCET pathway is substantially faster in the surface-immobilized case.

**Fig. 6:**
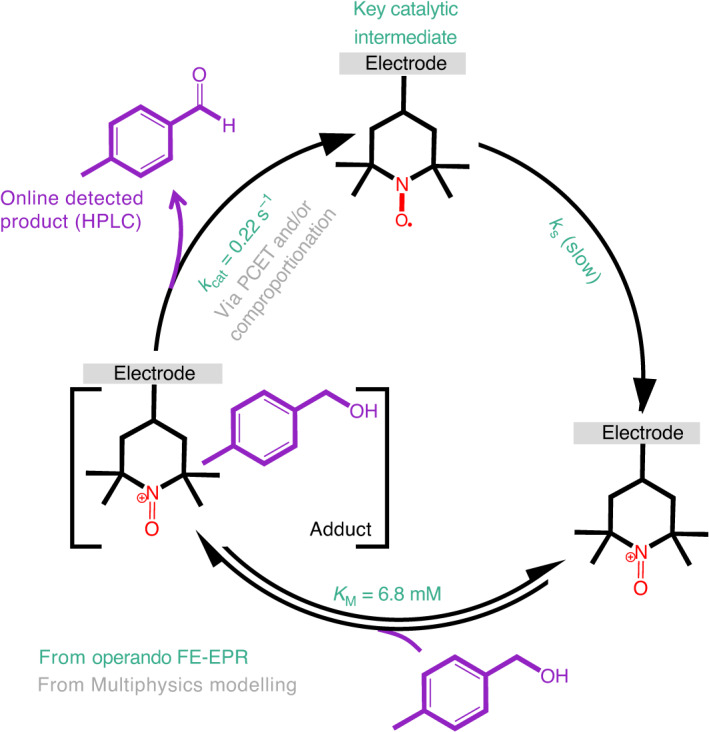
Overview of insights into alcohol oxidation catalysis by surface-immobilized nitroxide from operando FE-EPR. The mechanism of alcohol oxidation by surface-bound oxoammonium (yellow) is an EC′ mechanism described by the Michaelis–Menten model, with *k*_cat_ and *K*_M_ accessible from operando FE-EPR through the quantitative detection of the key STEMPO^•^ catalytic intermediate. *k*_s_ (also obtainable from electrochemistry alone) is slow, and the exact value is dependent on the electrode and experimental conditions, leading to *n*_app_ < 1 (Extended Data Fig. 7). The [Catalyst–Substrate] = [TEMPO^+^–RCH_2_OH] adduct formation is characterized by the Michaelis–Menten constant *K*_M_. Regeneration of the STEMPO^•^ catalyst in the steps following the rate-determining step (*k*_cat_) occurs via PCET and/or comproportionation (main text and equations (1) to (6)). Rate constants and *K*_M_ are given for the substrate MBA at pH 8.0 (Extended Data Figs. 8 and 9 present values for glycerol at pH 8.0 and MBA at pH 7.3, respectively).

Figure 6 summarizes the key findings from operando FE-EPR, made possible by (1) detecting a key active-site species (STEMPO^•^) during catalysis (Figs. 4 and 5) and (2) determining the timescales on which it is regenerated (Fig. 5). The combined structural information and kinetic resolution have led to a complete electrochemical model that can be interrogated to provide mechanistic insight. Although our investigations were dedicated to a surface-immobilized catalyst, the extensive body of work on solution-based nitroxides mediating alcohol oxidation (Extended Data Fig. 5 and Supplementary Section 4) provided inspiration and guided the elucidation of the mechanism, while omitting the challenges related to surface-immobilized catalysis resolved within. Thus, although further investigations of alcohol oxidation by surface-immobilized TEMPO will provide insights into the detailed mechanism, the kinetic resolution provided by operando FE-EPR made possible by detecting active-site catalytic intermediates has revealed the need to improve *k*_s_.

## Conclusion

The reported operando FE-EPR set-up enables the identification and kinetic resolution of paramagnetic intermediates formed by surface-immobilized redox catalysts. This economical, easy-to-replicate and user-friendly set-up has several advantages: (1) it is compatible with the widespread super-high-*Q* EPR cavities, (2) operation is possible at room temperature, with an aqueous solution and under flow conditions, and (3) a ‘true’ reference electrode (with a stable potential) can be used. These advantages make CV-based SEC measurements (FE_CV_-EPR) with excellent potential control possible. Besides exhibiting an electrochemical response on par with ‘pure’ electrochemical set-ups, our SEC experiments enable direct visualization of the electronic state of the catalyst (STEMPO) through in situ EPR measurements both under catalytic and non-catalytic conditions. Operando FE-EPR has enabled the measurement of essential kinetic parameters in this model surface-immobilized electrocatalytic reaction.

By using the experimentally determinable kinetic parameters from FE-EPR (*k*_s_, *k*_cat_ and *K*_M_), our electrochemical model could explain the observed trends in the catalytic wave shapes with increasing substrate concentrations (decreasing *n* and increasing *E*_catalytic_, as determined reliably with FE_CV_-EPR). This has revealed that the interplay between electron transfer and catalysis is key to the efficiency of the nitroxide catalyst. Although fast electron transfer is often assumed for small-molecular systems on surfaces, this is not the case, and the effect on the concentrations of catalytic intermediates within the catalytic cycle could be resolved. In the future, this experimental insight, combined with Multiphysics modelling to test and compare mechanistic hypotheses to the experimental data, could be extended to include the full toolkit of available electrochemical and EPR techniques. Freeze-quenching^16,19^ will open the prospect of investigating fast-relaxing radicals formed during catalytic reactions at any applied potential and using pulse EPR. Through the possibility to observe, identify and quantify low-concentration key paramagnetic active-site species and the ensuing kinetic resolution, operando FE-EPR has the potential to contribute to the rational design of catalysts by tuning immobilization strategies and local environments to achieve the transition to sustainable chemistry

## Methods

### Materials

All chemicals were purchased at analytical grade and used without further purification from Sigma-Aldrich. STEMPO was synthesized as described in ref. ^27^. ITO nanoparticles for making hierarchical electrodes were purchased from Sigma-Aldrich (nanopowder with particle size of <50 nm). Deionized ultrapure water (18 MΩ cm) was used for all experiments. The sodium carbonate buffer (Na_2_CO_3_, 500 mM) was adjusted to pH 8.0 or pH 7.3 with concentrated HCl_(aq)_. Stock solutions of 4-methylbenzyl alcohol (30 mM) and glycerol (5 M) were prepared in the Na_2_CO_3_ buffer.

### Electrode preparation and functionalization

Titanium strips (Sigma-Aldrich, thickness 0.127 mm, purity 99.7% trace metals) were cut to the dimensions shown in Fig. 2b and sonicated sequentially for 20 min in EtOH, followed by 2-isopropanol and finally acetone. ITO nanoparticles (5 wt%) were suspended in a pure ethanol solution with 5 M acetic acid. The nanoparticle solution was sonicated for 30 min, then 10 µl was drop-cast onto each side of the ITO-sputtered titanium strip. Once air-dried, the electrodes were heated from room temperature to 500 °C at a rate of 1 °C min^−1^, then annealed at 500 °C for 20 min. Once cooled, the working electrodes (henceforth referred to as mesoITO WEs) were cleaned by submersion in a mixture of 30% NH_4_OH:H_2_O:30% H_2_O_2_ (1:5:1 vol/vol) at 70 °C for 15 min, then dried in the oven at 180 °C for 1 h. STEMPO was immobilized on the electrode surface as described previously^27^.

### Film-electrochemistry

Electrochemical measurements were carried out using a µAutolabIII potentiostat in combination with NOVA software. Electrochemical experiments were performed using the standard three-electrode configuration, with the mesoITO WE, AgǀAgCl (3 M KCl) as the reference electrode (RE, World Precision Instruments) and a coiled nickel wire (thickness of 50 µm) as the counter electrode (CE). The electrolyte consisted of 500 mM Na_2_CO_3_ buffer (pH 8.0 or pH 7.3). Measurements were performed on the bench, open to the laboratory atmosphere, unless otherwise stated. Two electrochemical cells were used in this study: (1) a custom 3D-printed cell for FE-EPR applications (Fig. 2) and (2) a standard glass cell (Southampton University scientific glassblowing service) for validation of the FE-EPR set-up. All potentials were converted to the SHE. The shift of potential due to the *iR* drop (ohmic drop) was negligible and corrected after data collection (Supplementary Fig. 6).

### EPR spectroscopy

Continuous-wave X-band EPR measurements were performed at room temperature with an X-band Super High Sensitivity Probehead ER 4122SHQE unit (Bruker) and an EMX-T-DU/L Bruker spectrometer. To assess the performance of the electrochemical set-up and mesoITO WE before or after FE-EPR measurements, standard field-sweep EPR experiments were performed. The 2D field-delay experiments were carried out during FE-EPR measurements. EPR parameters for 2D field-delay experiments were chosen to minimize the sweep time of a single EPR spectrum while maximizing the resolution (62 mW microwave power, 10 mT sweep width, 2.6-s sweep time, 0.4 mT modulation amplitude, 100 kHz modulation frequency, 30 dB receiver gain and 1,000 points per scan).

### In situ FE-EPR cell

The lower canonical part of the FE-EPR cell was made of low-density polyethylene. The upper reservoir was 3D-printed with thermoplastic polylactic acid.

### In situ FE-EPR measurements

STEMPO-functionalized mesoITO WEs were rinsed with buffer to remove any unbound STEMPO and air-dried. Dry functionalized WEs were inserted into a standard EPR tube (Wilmad, 4-mm outer diameter clear fused-quartz (CFQ)) and an EPR field sweep was carried out (in the absence of any solvent) before FE measurements.

The FE-EPR set-up was assembled as follows: (1) the electrochemical cell was inserted into the EPR cavity; (2) the mesoITO strip was attached to a Ti wire (thickness, 10 µm) using parafilm; (3) the electrode was placed into the conical part of the FE-EPR cell (Fig. 2c); (4) circulation of 8 ml of Na_2_CO_3_ buffer (500 mM, pH 8) was initiated using a peristaltic pump (Pump P-1, General Electrics) operating at a rate of 30 mL h^–1^; (5) the Ag|AgCl RE was placed into the conical part of the FE-EPR cell as close as possible to the WE; (6) the Ni-wire CE was added to the reservoir of the FE-EPR cell; and (7) the electrodes were connected to the potentiostat. For the catalytic experiments, the substrate was added directly to the solution in the assembled cell inside the EPR cavity, by injecting the stock solution with the appropriate volume to give the final desired substrate concentration.

To perform in situ FE-EPR experiments under flow conditions, plastic tubing (inner diameter of 1 mm) was used to connect (1) the bottom part of the cell to the peristaltic pump and (2) the peristaltic pump to the upper part of the cell, as shown in Fig. 2. The flow direction was from the top to the bottom of the cell, with the flow speed controlled by the peristaltic pump. The part of the plastic tubing inside the EPR cavity was inserted into a 4-mm open-ended quartz tube (Fig. 2) to avoid distortion and prevent any leakage from entering the cavity. Insertion of the mesoITO strip into the SHQE EPR cavity led to a 40% drop in its quality factor *Q*. Field-delay EPR experiments were launched simultaneously with starting the CV scan and stopped at the end of the electrochemical experiment.

For the FE_CV_-EPR experiments, 2D field-delay EPR was launched with a duration of 4.1 s per EPR spectrum (2.6-s sweep time + 1.5-s processing time) while sweeping the potential at 5 mV s^−1^, which led to steps of 21 mV per EPR spectrum. Under these conditions, the total duration of the FE_CV_-EPR experiment was ~340 s.

For the FE_amp_-EPR experiments, the potential was held at +0.6 V such that only the EPR-active species (STEMPO^•^) was present, then stepped to +1.1 V such that the EPR-silent species (STEMPO^+^) was generated exclusively. The current was monitored at OCP over time and the experiment was performed with and without substrate. EPR spectra were recorded throughout the chronoamperometry-OCP measurement.

### Morphological characterizations

Scanning electron microscopy (SEM) images were collected using a Zeiss LEO Gemini 1525 field-emission-gun scanning electron microscope (FEG-SEM) with an InLens detector, using an accelerating voltage of 5 kV and 30-µm standard aperture. The top surface images of the mesoITO electrode were taken by fixing the electrode to a standard flat SEM specimen stub. The cross-sectional images were taken by fixing the electrode to a 45°-angled SEM specimen stub.

Confocal topography analysis was conducted using a Zeiss LSM 800 laser confocal scanning microscope with ZEN Blue 2.6 software. Confocal reflection-mode image stacks (*Z*-stack interval of 0.26 µm) were obtained using a 405-nm laser (2.0% power) with a ×20/NA 0.7 objective lens. The collected stacks were then processed using ConfoMap software to obtain the 3D view image and determine the film thickness of the mesoITO electrode.

### HPLC for product analysis and quantification

During the stepped chronoamperometry catalytic reaction, a 1-ml aliquot of the solution was taken from the FE-EPR cell at the end of each potential step. The aliquot was then analysed using an Agilent 1260 Infinity II HPLC system and a diode array detector monitoring 254 nm (Supplementary Fig. 20). A 2-µl volume of the sample was injected into a flow of 0.5 ml min^−1^ through a 50 mm × 2.1 mm Raptor C18 column (particle size, 2.7 µm) purchased from Restek. The temperature was 40 °C and the mobile phase consisted of a mixture of acetonitrile (MeCN) and 5 mM aqueous ammonium formate, with a gradient from 5% MeCN to 95% MeCN over 1 min, followed by a hold at 95% MeCN for 3.5 min. Standard calibration curves for 4-methylbenzaldehyde (Supplementary Fig. 21) were then generated to quantify the product and determine the concentration of species in the reaction aliquot.

### Data analysis

#### CV analysis by the Laviron method

The Laviron analysis was carried out using the following equations:

7 $${\Delta {E}_{{\rm{p}},\,{\rm{a}}}=\frac{-2.3{RT}}{\left(1-\alpha \right){nF}}\log \left(\frac{\left(1-\alpha \right){nF}}{{RT}{k}_{\rm{app}}}\right)-\frac{2.3{RT}}{\left(1-\alpha \right){nF}}\log \left(\nu \right)}$$

8 $${\Delta {E}_{{\rm{p}},\,{\rm{c}}}=\frac{-2.3{RT}}{\alpha {nF}}\log \left(\frac{\alpha {nF}}{{RT}{k}_{\rm{app}}}\right)-\frac{2.3{RT}}{\alpha {nF}}\log \left(\nu \right)}$$

where Δ*E*_p, a_ and Δ*E*_p, c_ are the differences between the potential of the anodic (a) and cathodic (c) peaks to the formal reduction potential (*E*_non-catalytic, CV_) obtained by averaging the anodic and cathodic potentials at 5 mV s^−1^, *n* is the number of electrons transferred, *α* is the electron-transfer coefficient, *ν* is the scan rate, and *k*_app_ is the apparent rate constant for electron transfer. *R*, *T* and *F* are the ideal gas constant, absolute temperature and Faraday constant, respectively.

The Laviron method (Supplementary Fig. 4) was applied by linearly fitting the anodic and cathodic regions of the trumpet plot for values of Δ*E*_p, a_, Δ*E*_p, c_ > 100 mV. (1 − *α*) and *α* were determined from the gradients of the anodic and cathodic trends respectively. *k*_app, a_ and *k*_app, c_ were deduced from the *y* intercept, and the critical scan rate, *υ*_c_, was obtained from the *x* intercept by extrapolating the linear regression of the trumpet plot in both regions to Δ*E*_p_ = 0.

#### EPR signal intensity calibration

The concentration of STEMPO radical observed by EPR was determined through calibration with different known concentrations of 4-aminoTEMPO. The same electrode configuration was used in the FE-EPR cell as in Fig. 2a, but instead of attaching STEMPO to the WE surface, 4-aminoTEMPO was added to the solution at different concentrations. The resulting linear dependency (Supplementary Fig. 8) of the 4-aminoTEMPO EPR signal intensity versus concentration was then used to determine the concentration of STEMPO radicals observed in FE_amp_-EPR measurements. STEMPO^•^ concentrations of 80 and 70 µM were determined on the electrodes used for measurements with MBA and glycerol, respectively.

#### Michaelis–Menten model

The Michaelis–Menten model was used to fit the catalytic currents and radical regeneration rates, based on the Michaelis–Menten equation:

9 $${\upsilon =\frac{{V}_{\max }[{\rm{Substrate}}]}{{K}_{{\rm{M}}}+[{\rm{Substrate}}]}}$$

where *v* is the rate of the reaction, *V*_max_ is the maximum rate of the reaction, and *K*_M_ is the Michaels–Menten constant. *V*_max_ and *K*_M_ were deduced from the plots obtained. The binding affinity is the inverse of the Michaelis–Menten constant. The catalytic rate *k*_cat_ was calculated using the following equation:

10 $${k}_{{\rm{cat}}}={\frac{{V}_{\max }}{{[{\rm{Catalyst}}]}_{\rm{i}}}}$$

where [Catalyst]_i_ is the initial concentration of catalyst, here STEMPO, quantified from EPR (Supplementary Fig. 8 and Supplementary Section 7).

#### Nernst fitting of FE-EPR data

The Nernst equation was used to fit the FE-EPR data for the ${{\rm{STEMPO}}}^{+}+n{\rm{e}}^{-}\rightleftharpoons {{\rm{STEMPO}}}^{{\rm{\bullet }}}$ equilibrium reaction:

11 $${E}={E}^{\circ }+\frac{{{RT}}}{n{{F}}}\mathrm{ln}\frac{[{{\rm{STEMPO}}}^{+}]}{[{{\rm{STEMPO}}}^{{\rm{\bullet }}}]}$$

where *E* is the potential in the cell, ${E}^{\circ }$ is the standard reduction potential, *R* is the gas constant, *T* is the temperature (here room temperature), *n* is the number of electrons transferred, and *F* is the Faraday constant.

Knowing that only STEMPO^•^ is EPR-active, and that at low potentials, all STEMPO species are in the STEMPO^•^ form, the total concentration of STEMPO is equal to [STEMPO^•^]_i_. Consequently, [STEMPO^•^]_i_ *=* [STEMPO^+^] + [STEMPO^•^].

[STEMPO^•^] is equivalent to the double integral of the STEMPO radical EPR signal. At room temperature, this leads to the following equation used to plot FE_CV_-EPR data:

12 $${\iint {{\rm{STEMPO}}}^{{\rm{\bullet }}}=\frac{\iint {{{\rm{STEMPO}}}^{{\rm{\bullet }}}}_{\rm{i}}}{1+{10}^{\frac{n\left(E-{E}^{\circ }\right)}{0.06}}}}$$

where ${\iint {{\rm{STEMPO}}}^{{\rm{\bullet }}}}$ are the double integrals of the STEMPO^•^ EPR spectra.

#### CV FE-EPR data analysis

To process the acquired electrochemical and EPR data, we developed a MATLAB-based program. This program plots EPR intensities or spin concentrations as a function of the applied potential with fitting to the Nernst equation. The analysis procedure for the FE_CV_-EPR data is shown in Supplementary Video 2 and summarized here briefly. The EPR and CV data were first plotted independently. If multiple CV scans were performed, one CV scan (with the corresponding EPR data) was selected for the subsequent analysis. Because the time axis is the common basis between the EPR and CV data, the chosen CV scan and the corresponding EPR data were split into forward and reverse datasets. The forward and reverse EPR data were baseline-corrected using the function basecorr in EasySpin^77^ before double integration. To assign a potential value to each EPR spectrum acquired during the forward CV scan, the potential range corresponding to the forward CV scan was divided by the total number *x* of EPR spectra of this range. The resulting potential increment was used as a ‘step’ on the forward CV potential axis, resulting in *n* different points on this axis, with each potential value corresponding to one EPR spectrum. The double integrals of the EPR spectra (*y* axis) were plotted as a function of potential obtained from the forward CV scan (*x* axis) and fitted to the Nernst equation. The Nernst fit was optimized directly using the Simplex derivative-free method through the fminsearch MATLAB function. The same process was repeated for the reverse CV scan datasets.

#### Chronoamperometry-EPR data analysis

Double integrals of the EPR spectra (following baseline correction, as described for the CV-EPR data) were plotted against time. The resulting data, corresponding to the regeneration of the nitroxide radical, were fitted using first-order relaxation:

13 $${y={{A}_{1}{\rm{e}}}^{\left(-t\times k\right)}+{y}_{0}}$$

where *y* is the EPR double integral or radical concentration, *A*_1_ is the amplitude, *t* is the time, which corresponds to the *x* axis, *k* is the rate of regeneration of the radical and *y*_0_ is the asymptotic constant reached when *t* → ∞.

#### Multiphysics modelling

COMSOL Multiphysics 6.1 with electroanalysis and laminar flow modules was used for Multiphysics modelling to describe the solution (electro)chemical reactions and convection, respectively. This model solves ordinary differential equations corresponding to well-defined analytical expressions of mass transport, (electro)chemical reaction rates and equilibria. Further details are provided in Supplementary Section 9.

## Online content

Any methods, additional references, Nature Portfolio reporting summaries, source data, extended data, supplementary information, acknowledgements, peer review information; details of author contributions and competing interests; and statements of data and code availability are available at 10.1038/s41557-024-01450-y.

## Supplementary information

Supplementary Information Surface characterization of the mesoITO working electrode, electrochemical and EPR characterization of the FE-EPR cell, the mechanism of solution-based electrocatalytic alcohol oxidation by nitroxides, the general saturation behaviour model, production detection, electrochemical analysis, foot of the wave analysis, Supplementary Figs. 1–28 and Tables 1–6.

Supplementary Video 1 Example CV scan measured at 5 mV s^−1^, with a selected set of corresponding EPR spectra.

Supplementary Video 2 Analysis procedure of FE_CV_-EPR data.

## Source data

Source Data Fig. 2 Unprocessed confocal image.

Source Data Fig. 2 Unprocessed SEM image.

Source Data Fig. 3 Electrochemistry and EPR source data from FE(CV)-EPR experiments.

Source Data Fig. 4 Electrochemistry and EPR source data from FE(CV)-EPR experiments.

Source Data Fig. 5 FEamp-EPR data.

Source Data Extended Data Fig./Table 1 Unprocessed electrochemistry data.

Source Data Extended Data Fig./Table 2 EPR data in Excel format.

Source Data Extended Data Fig./Table 3 EPR data in Excel format.

Source Data Extended Data Fig./Table 4a Modelling data.

Source Data Extended Data Fig./Table 4a Modelling data.

Source Data Extended Data Fig./Table 4c Modelling data.

Source Data Extended Data Fig./Table 6 EPR data in excel format

Source Data Extended Data Fig./Table 7a Modelling data.

Source Data Extended Data Fig./Table 7b Modelling data.

Source Data Extended Data Fig./Table 8 FEamp-EPR data.

Source Data Extended Data Fig./Table 9 FEamp-EPR data.

Source Data Extended Data Fig./Table 10 Modelling data.

## Data Availability

Data for the main text and Supplementary Information are available from the Imperial Research Data repository (10.14469/hpc/13519). Source data are provided with this paper.

## Code availability

Custom MATLAB-based FE-EPR data analysis programs to accompany the roadmap for data analysis provided in Supplementary Videos 1 and 2 are available from the Imperial Research Data repository (10.14469/hpc/13517). COMSOL Multiphysics models were built based on the information provided in Supplementary Section 9. Model reports are available from 10.14469/hpc/13517.
