# Supplementary material for: Operando film-electrochemical EPR spectroscopy tracks radical intermediates in surface-immobilized catalysts
Source: Nat Chem. 2024 Feb 14;16(6):1015–23. doi: 10.1038/s41557-024-01450-y (PMC11636982; doi:10.1038/s41557-024-01450-y)
Supplement: Supplementary file 1 — Surface characterization of the mesoITO working electrode, electrochemical and EPR characterization of the FE-EPR cell, the mechanism of solution-based electrocatalytic alcohol oxidation by nitroxides, the general saturation behaviour model, production detection, electrochemical analysis, foot of the wave analysis, Supplementary Figs. 1–28 and Tables 1–6. [file 41557_2024_1450_MOESM1_ESM.pdf]

nature chemistry

Article https://doi.org/10.1038/s41557-024-01450-y

Operando film-electrochemical EPR spectroscopy tracks radical intermediates in surface-immobilized catalysts

In the format provided by the authors and unedited

Table of Contents

1. Surface characterisation of the mesoITO working electrode 2

2. Electrochemical characterisation of FE-EPR cell 2

Current response of mesoITO working electrode 2
Performance in a standard electrochemical cell 2
Effect of flow direction 3
Quantification of Ohmic drop 4
Comparison of CV characteristics in the FE-EPR cell vs a standard electrochemical cell 5
Anaerobic vs aerobic conditions 6

3. EPR characterisation of FE-EPR cell 7

EPR performance – microwave power saturation 7
EPR performance – sweep time 7
EPR signal calibration 8

4. Mechanism of solution-based electrocatalytic alcohol oxidation by nitroxides as described in the literature 9

5. Supporting data for FE_{CV}-EPR experiments 10

Nernst fits for different MBA and glycerol concentrations 10
Substrate-concentration dependent *E* shifts under aerobic and anaerobic conditions 12

6. Supporting data for FE_{amp}-EPR experiments 13

Non-catalytic experiments under anaerobic conditions and at different pH values 13
During catalysis with MBA under aerobic conditions (pH 8.0) 14
During catalysis with MBA as substrate under anaerobic conditions 15
During catalysis with glycerol under aerobic conditions 16
During catalysis with MBA as substrate under aerobic conditions (pH 7.3) 17
Reproducibility 18
Summary of thermodynamic and kinetic parameters 18

7. Michaelis-Menten kinetics in context with the general saturation behaviour model 19

8. Product detection 20

9. Electrochemical analysis 22

Multiphysics Model Overview 22
Electrolyte 22
Wall boundary conditions 23
Inlet 24
Outlet 24
Electrode Surface 24
Supplementary Figures for Multiphysics modelling 26
Supplementary Tables for Multiphysics modelling 29

10. Foot of the wave analysis 30

11. References 31

1. Surface characterisation of the mesoITO working electrode

Figure 1 shows scanning electron microscopy (SEM) images of the mesoporous ITO (mesoITO) electrode of the top surface at different magnitudes (left and middle) and a cross-section (right image). See Methods for further details. The mesoITO thickness was determined to be 2.3 ± 0.4 µm from SEM, in agreement with 2.6 ± 0.2 µm from confocal microscopy.

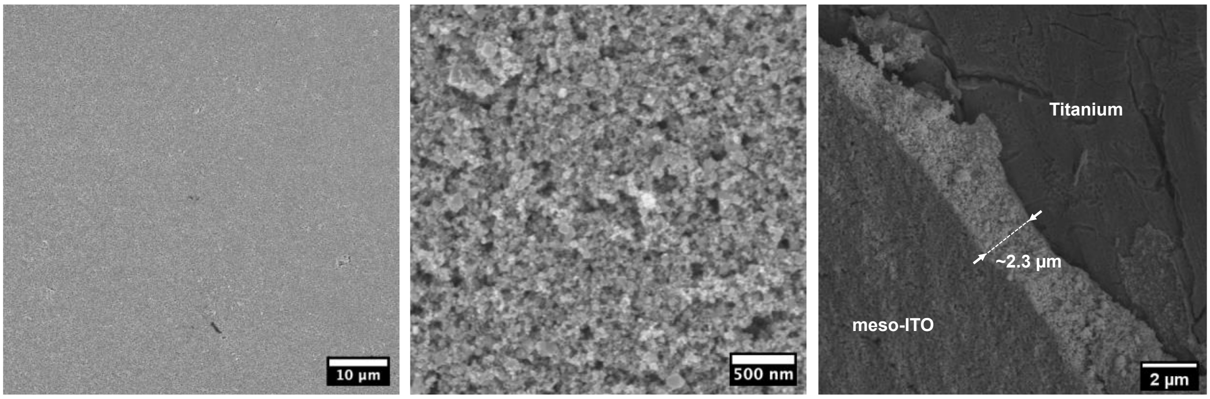

Figure 1. SEM images of the mesoITO electrode surface.

2. Electrochemical characterisation of FE-EPR cell

Current response of mesoITO working electrode

The change of the current density as a function of time when stepping the potential from 0 mV to 500 mV was measured on a bare mesoITO (Figure 2a) and an electrode with STEMPO attached (Figure 2b). The time required for the current to return to equilibrium is 0.3 s on the bare electrode and 2 s when STEMPO is attached to the electrode.

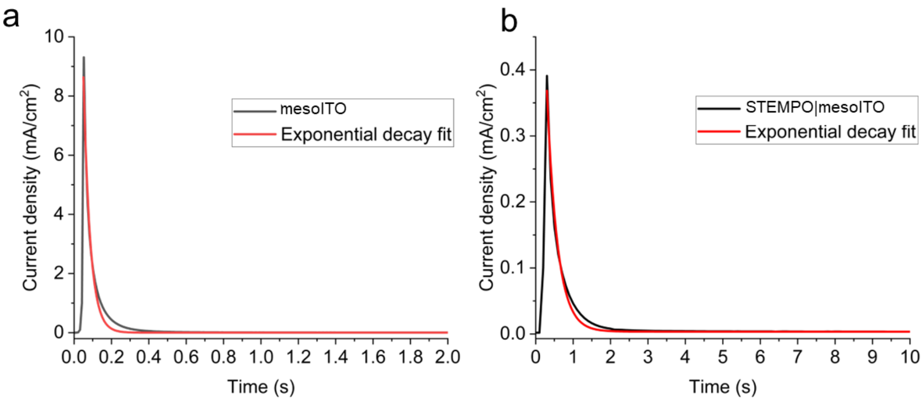

Figure 2. Current response profile of the mesoITO electrode. a, the current response was recorded after stepping the potential from 0 mV to 500 mV vs SHE on a bare mesoITO electrode. **b**, corresponding current response of STEMPO|mesoITO. The red fit corresponds to a first order exponential decay.

Performance in a standard electrochemical cell

The cyclic voltammogram of $STEMPO^{\bullet}/STEMPO^{+}$ attached to the mesoITO electrode recorded in a standard electrochemical cell at a scan rate of 10 mVs^{-1} is shown in Figure 3. CVs at different scan rates were recorded under the same conditions (Figure 4a).

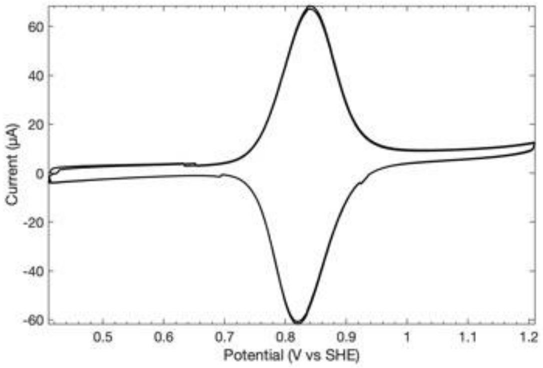

Figure 3. Cyclic voltammogram of STEMPO• attached to a mesoITO electrode recorded in a standard electrochemical cell. Experimental conditions were 10 mVs^{-1} scan rate, 500 mM Na_2CO_3 buffer, pH 8.0, 20 °C.

Non-turnover peaks of the $STEMPO^{•}/STEMPO^{+}$ redox couple show a reversible electron transfer reaction with a peak-to-peak separation of 20 mV (Fig. 3) and a reduction potential of +830 mV vs SHE, in agreement with previously reported values[1]. The full width at half maximum is 100 mV (Fig. 3) and points toward a one electron transfer process[1]. The electron-transfer properties were further characterized using the Laviron method[2], with the critical scan rate determined to be υ_c = 71 mVs^{-1} and the apparent electron transfer rate constant k_{app} = 0.77 s^{-1} (Fig. 4b). At scan rates lower than υ_c, the linear relationship between peak current and scan rate showed that STEMPO was surface bound (Fig. 4c).

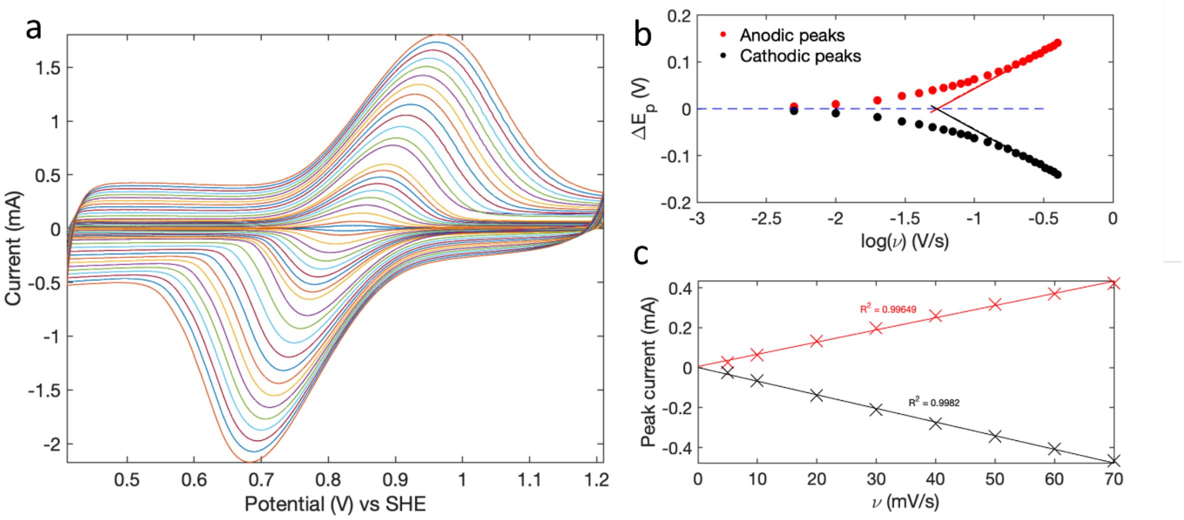

Figure 4. Electrochemical characterization of the mesoITO electrode. a, Cyclic voltammograms showing oxidoreduction of $STEMPO•/STEMPO^{+}$ recorded at different scan rates from 400 mVs^{-1} to 5 mVs^{-1}. **b**, Trumpet plot deduced from the variable scan rate CV measurements in a. **c**, i_p vs v plot for $v < v_c$. Measurements were performed in a standard glass electrochemical cell using Ag|AgCl as RE, nickel wire as CE and an aqueous solution of 500 mM Na_2CO_3, pH 8.0 at 20°C. The apparent electron transfer rate, k_s = 0.77 s^{-1}, was calculated using the Laviron equations (see Methods, equations 1 and 2). The critical scan rate, υ = 71 mVs^{-1}, was deduced from the x-intercept of the intersection of the linear regression of both cathodic and anodic potentials shifts (Fig. 4b).

Effect of flow direction

All the measurements in this study are performed by flowing the electrolyte from the top to the bottom of the cell. To ensure that the direction of the flow does not affect the electrochemical

data, we recorded CVs with the opposite flow direction (Figure 5). No difference in peak current, peak separation and overall CV shape was observed between the two flow directions.

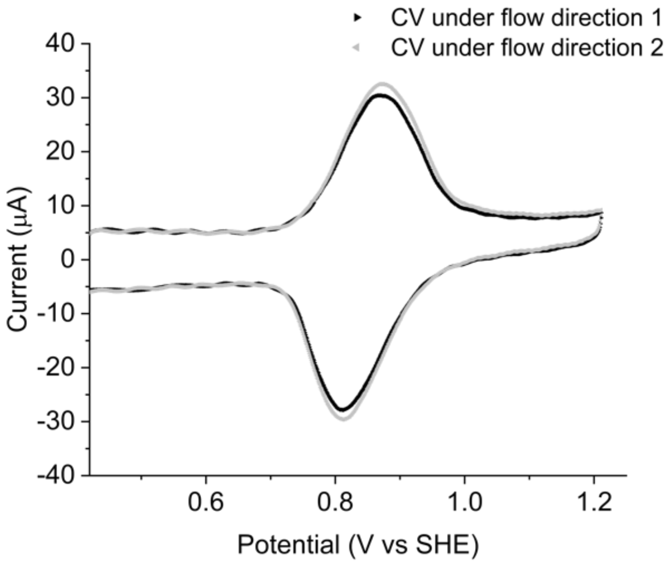

Figure 5. Effect of flow direction. CVs of STEMPO•/$STEMPO^+$ oxidoreduction performed in FE-EPR cell under flow conditions. Direction 1 (black) represents the flow from the top to the bottom of the cell and direction 2 (grey) describes the flow from the bottom to the top of the FE-EPR cell. Scan rate 5 mVs^{-1}.

Quantification of Ohmic drop

To calculate the Ohmic (*i*R) drop and determine the shift it is causing to the potential, the solution resistance between the working and the reference electrodes was determined by electrochemical impedance spectroscopy (EIS). EIS was performed in the FE-EPR cell (Figure 6a) with the reference electrode positioned at the top part of the ITO structure of the working electrode outside of the EPR cavity (Figure 6b), as during all SEC measurements in the FE-EPR cell. This relative position of the reference electrode was unchanged throughout the study. The EIS measurements were carried out under flow conditions and the results from the two directions are in almost perfect agreement (Figure 6c). A wide range of circuits led to a satisfactory fit of the experimental data (not shown). Importantly, the solution resistance in all circuits, a common element in the tested circuits, was almost constant, ranging from 19 to 34 Ω with an average of 27 Ω. With this average resistance value and the non-catalytic (and later catalytic) current, the potential was corrected by subtracting $i*R_s$ (R_s = solution resistance) from the potential read-out on the potentiostat during a given CV.

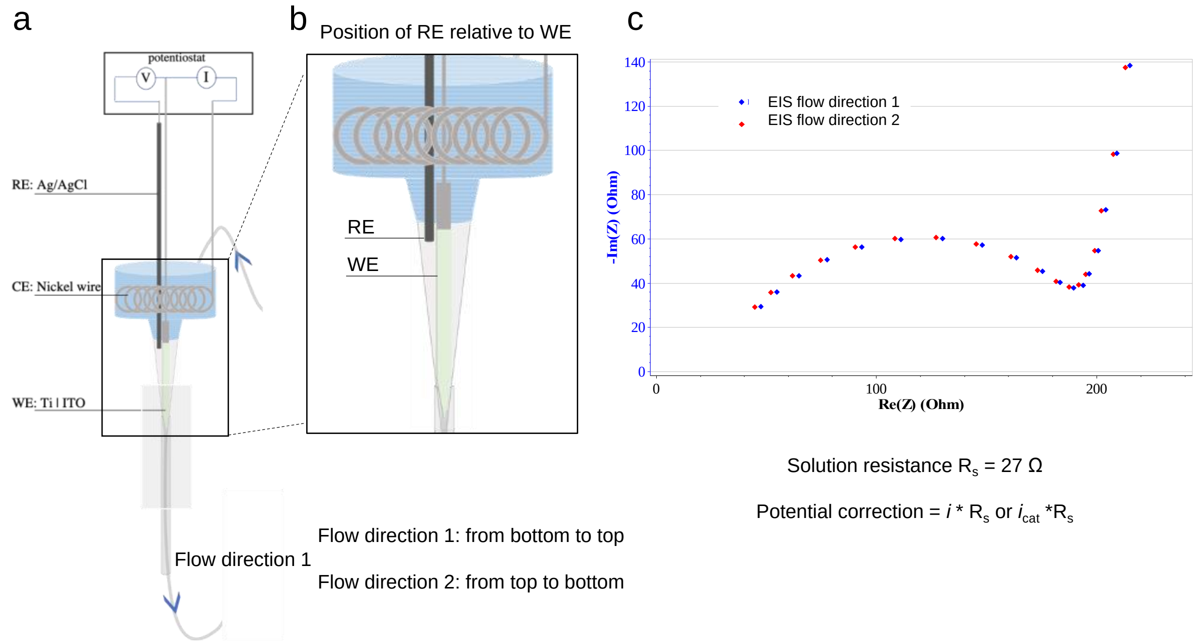

Figure 6. EIS measurements of the FE-EPR cell under flow conditions. a, FE-EPR cell and electrode configuration reflecting the configuration of all the experiments performed in this study. **b**, Relative position of the RE to the WE. **c**, EIS measurements under flow in direction 1, from top to the bottom of the cell, and direction 2, from bottom to the top of the cell. The solution resistance (R_S) found in both cases was 27 Ω.

Comparison of CV characteristics in the FE-EPR cell vs a standard electrochemical cell

Table 1. Summary of CV characteristics of STEMPO$^{\bullet}$/STEMPO^{+} couple in a standard electrochemical cell vs our flow FE-EPR cell. The data in a standard electrochemical cell is taken from Supplementary Figures 3 and 4 and the data in the FE-EPR under flow conditions is taken from Extended Data Figure 1 and main text Figure 3.

	$E_{STEMPO\bullet/STEMPO+}$ (mV vs SHE)	Peak-to-peak separation at 10 mV s^{-1} (mV)	FWHM at 10 mV s^{-1} (mV)
Standard electrochemical cell	830	20	100
FE-EPR flow cell	830	19	105

Anaerobic vs aerobic conditions

All experiments presented in this study were performed in the presence of atmospheric O_2. To ensure that the FE-EPR data acquired were not affected by O_2, we performed *in situ* electrochemical measurements (Figure 7) as well as FE_{CV}-EPR and FE_{amp}-EPR measurements (Figures 12 and 13a/15, respectively) while purging the FE-EPR cell with house N_2. CVs of the same STEMPO-functionalised electrode recorded under anaerobic and aerobic conditions, under flow, are shown in Figure 7. The $STEMPO^{\bullet}/STEMPO^{+}$ non-turnover peak is visible at +830 mV vs SHE unchanged in both cases. The anaerobic CV further reveals the non-turnover peaks corresponding to the $STEMPO^{\bullet}$/STEMPOH redox couple with a reduction potential of -250 mV vs SHE much more clearly than the aerobic CV, because these are no longer masked by O_2 reduction.

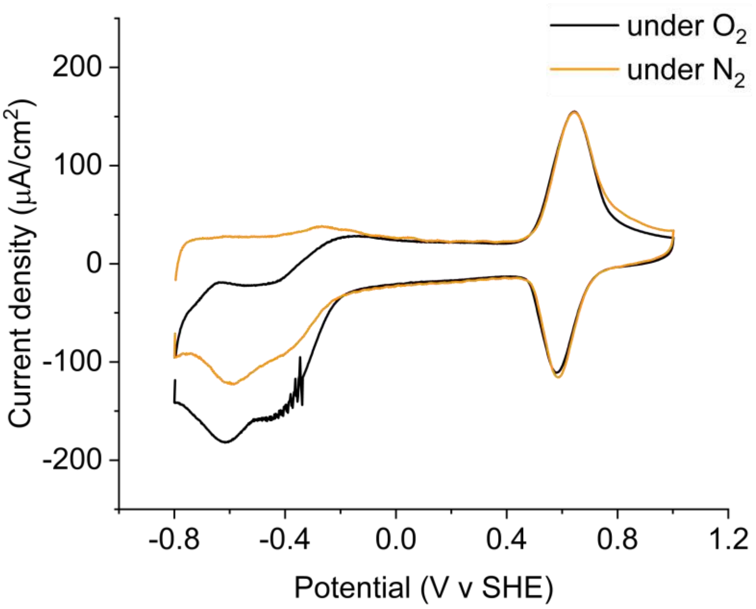

Figure 7. Comparison of CVs of STEMPO|mesoITO electrodes under aerobic and anerobic conditions. Experiments were performed on the same electrode in the FE-EPR cell, with 8 ml of 500 mM Na_2CO_3 buffer at pH 8.0. Scan rate 5 mVs^{-1}. For anaerobic experiments, nitrogen gas was purged directly into the cell *in situ* during FE-EPR measurements.

3. EPR characterisation of FE-EPR cell

EPR performance – microwave power saturation

The EPR signal of surface-bound TEMPO (in the FE-EPR cell in aqueous buffer) is asymmetric (Extended Data Fig. 2). This is the result of the asymmetric distribution of the B_1 field in the EPR cavity, due to inserting lossy material into the top half of the resonator. The corresponding EPR spectra were found to exhibit unusually high non-saturating microwave powers (Figure 8), which we attribute to absorption of microwave energy by the high surface area WE material and aqueous buffer.

Figure 8 shows the microwave saturation profile of the nitroxide attached to the mesoITO electrode placed in pH 8.0 buffer solution in the FE-EPR set-up and inserted into the EPR cavity as shown in Figure 2 of the main paper. Above 64 mW the double integrals of the nitroxide EPR signal deviate from the linear regime (Figure 8). Consequently, 64 mW (the highest non-saturating microwave power) was used to perform all FE-EPR measurements in this study.

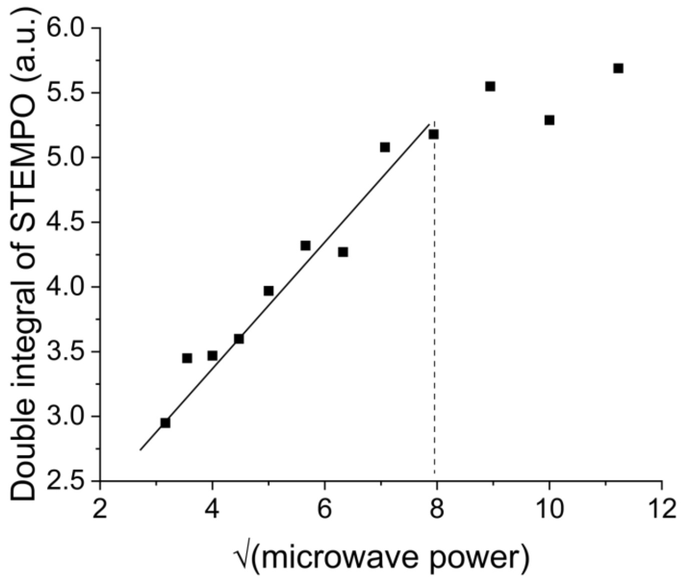

Figure 8. Saturation behaviour of STEMPO• in the FE-EPR cell. Microwave saturation curve of STEMPO• in the FE-EPR cell measured in an SHQE cavity at room temperature showing non-saturating (grey) and saturating (pink) regions.

EPR performance – sweep time

The EPR spectrum of STEMPO attached to a mesoITO electrode recorded with 50 s sweep time is shown in Figure 9 (black line). Reducing the sweep time to 2.6 s (to enable FE_{CV}-EPR measurements) resulted in a 0.5 mT shift of the magnetic field without affecting the intensity, shape and linewidth of the signal (Figure 9).

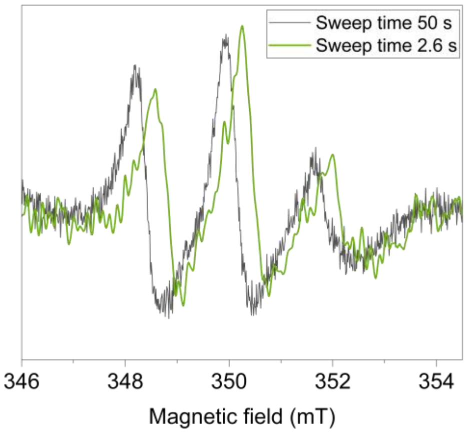

Figure 9. Effect of sweep time on EPR spectra. EPR spectra are shown for the short sweep time (green) used throughout this work, compared to a more standard sweep time (black). Other EPR acquisition parameters were as follows: 64 mW microwave power, 10 mT sweep width, 0.4 mT modulation amplitude at 100 kHz modulation frequency, 30 dB receiver gain, 1000 points per scan.

EPR signal calibration

To quantitatively determine the STEMPO radical concentration during FE-EPR experiments, a calibration was carried out with 4-amino TEMPO. The FE-EPR cell (electrode configuration as shown in Figure 2 (main manuscript), with an unfunctionalised mesoITO working electrode) was filled with different 4-aminoTEMPO solutions of known concentration. EPR measurements were then carried out under non-saturating conditions leading to the calibration curve shown in Figure 10.

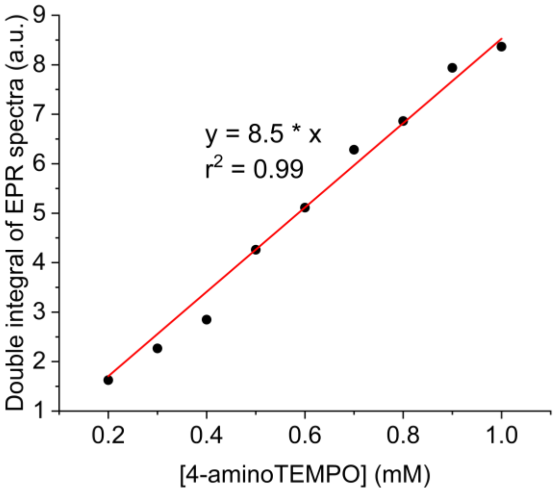

Figure 10. Calibration curve to quantify the STEMPO$^{\bullet}$ formation. Double integrals of the EPR spectra of 4-aminoTEMPO solutions of known concentration dissolved in 500 mM Na_2CO_3 buffer in FE-EPR cell, with the same configuration used throughout this study. EPR measurement conditions were as follows: 64 mW microwave power, 0.4 mT modulation amplitude at 100 kHz modulation frequency, 30 dB receiver gain.

4. Mechanism of solution-based electrocatalytic alcohol oxidation by nitroxides as described in the literature

In this section we summarise briefly the *solution-based* electrocatalytic mechanism of alcohol oxidation by nitroxyl radicals, as described in the literature (see Extended Data Fig. 5). The radical is electrochemically oxidised to generate the catalytically active oxoammonium species $TEMPO^+$ (red box in Extended Data Fig. 5). A pre-oxidation step then involves the formation of an oxoammonium-alcohol adduct by nucleophilic attack to the nitrogen atom of oxoammonium (light blue box) and is supported by mechanistic studies[3–6].

The prevailing route for the dissociation of the adduct, leading to the oxidation of the alcohol to the corresponding carbonyl, involves the formation of hydroxylamine by a rate-determining intramolecular proton transfer from the alcohol to the oxoammonium (route II)[6–8]. However, hydroxylamine was not detected experimentally and the only experimental study investigating the fate of oxoammonium after catalysis led to the indirect detection of a radical instead of hydroxylamine[9]. To account for this observation, the authors of this study proposed a mechanism that does *not* include hydroxylamine in the catalytic states, instead suggesting a pre-equilibrium between alcohol and base prior to the oxidation reaction (route II'). Mechanistic proposals for the catalyst regeneration (green box) assume the formation of hydroxylamine during catalysis, with two routes proposed for the regeneration of the oxoammonium: comproportionation (route III) followed by one-electron oxidation reaction (IV) or $2e^-/1H^+$ proton-coupled electron transfer, PCET (route V)[6,7]. Comproportionation is proposed to be more thermodynamically and kinetically favourable in solution, while PCET is more energetically demanding[10,11]. The high ratio of alcohol:nitroxyl leads to a high oxoammonium consumption rate and consequently a decrease in the rate of comproportionation, since this requires the copresence of both oxoammonium and hydroxylamine[10,11]. To compete with PCET at high substrate concentrations the comproportionation rate must exceed 10^5 $mol^{-1}s^{-1}$, as deduced from CV simulations[10].

5. Supporting data for FE_{CV}-EPR experiments

Nernst fits for different MBA and glycerol concentrations

Double integrals of EPR spectra from FE_{CV}-EPR measurements are performed aerobically under catalytic conditions by addition of different concentrations of MBA (Figure 11a) glycerol (Figure 11b). For example, the traces in Figure 11a (left panel) corresponds to the data shown in Figure 4d in the main paper. Fits to the Nernst equation are shown as solid lines. All MBA measurements were performed on one single electrode. Measurements with glycerol were also performed on a single electrode. The substrate concentration was increased progressively through addition of a concentrated substrate solution (with negligible effect on the total volume).

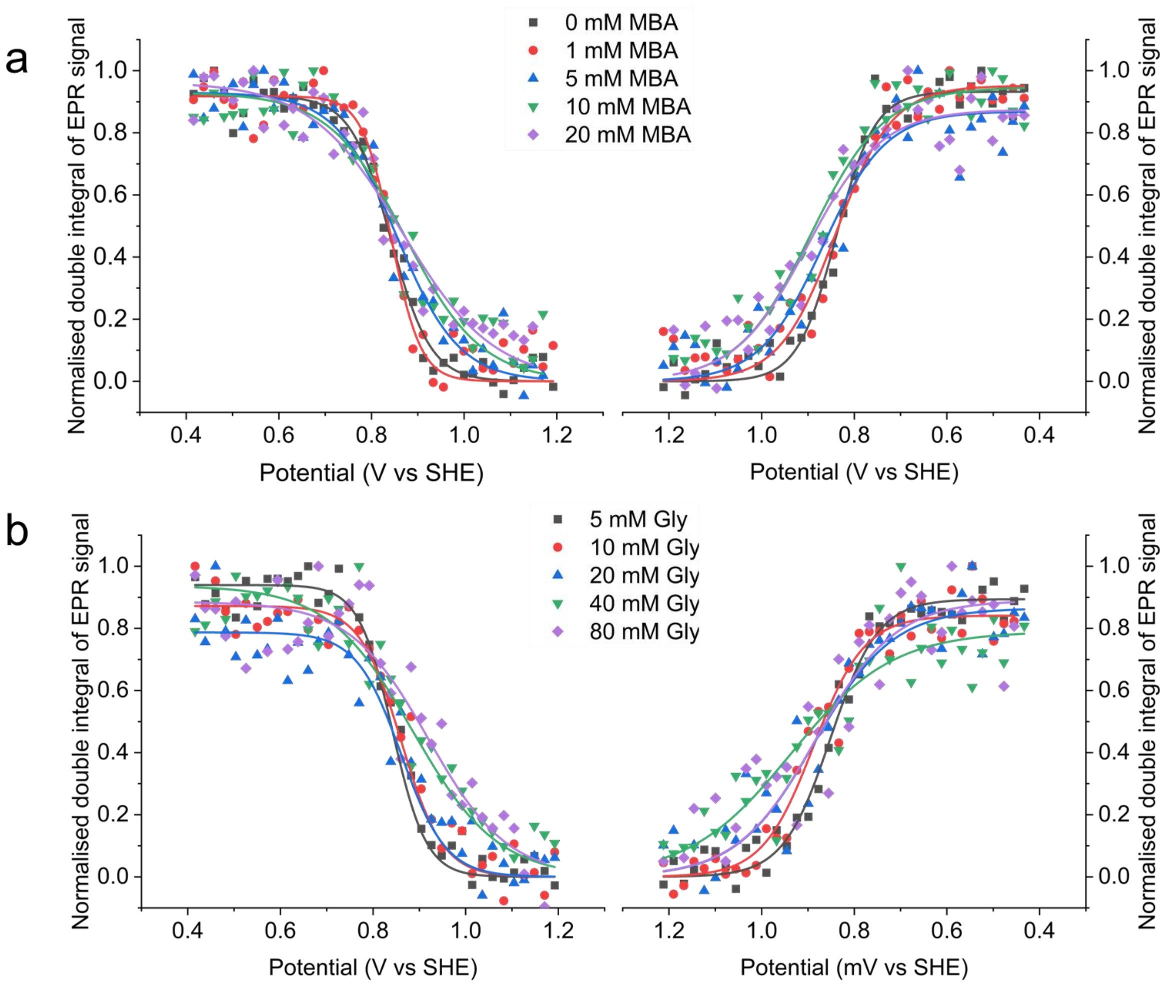

Figure 11. Nernst fits of EPR data for different concentrations of MBA and glycerol (pH 8.0). Normalised double integrals of EPR spectra of STEMPO$^{\bullet}$ recorded during the forward (left) and reverse (right) scans of the *in situ* catalytic CV performed with different concentrations of: **a,** MBA and **b,** glycerol, fitted with the one-electron Nernst equation (solid lines). See Table 2 for fit parameters.

Excellent agreement is obtained between $E_{catalytic(FE\text{-}EPR)}$ and $E_{catalytic(CV)}$ for both substrates at different concentrations (Table 2). Film loss[9] therefore did not affect data interpretation and the conclusions drawn from this study. n_{app} (which is accessible from the FE-EPR data but not from the CVs alone) decreases with increasing substrate concentration (Table 2), and this is in full agreement with the Multiphysics model (Section 9). The catalytic reduction potentials

($E_{catalytic}$) of STEMPO$^{•}$/STEMPO^{+} deduced from the CVs and the potentials deduced from Nernst fitting the EPR data with n_{app} considered in the Nernst equations are given in Table 2.

Table 2. Summary of the catalytic parameters deduced from FE$_{CV}$-EPR experiments with different substrate concentrations (pH 8.0). Ohmic drop correction of $E_{(catalysis,\ CV)}$ was carried as described in Figure 6.

**Substrate**	**Concentration** mM	$E_{(catalytic,\ CV)}$ mV	$E_{(catalytic,\ CV)}$ –*iR*corrected mV	$E_{(catalytic,\ FE\text{-}EPR)}$ mV	n_{app}
**MBA**	1	849	848	842 ±5	0.61 ±0.06
	5	866	864	862 ±6	0.38 ±0.03
	10	874	872	883 ±7	0.31 ±0.03
	20	881	879	883 ±8	0.29 ±0.02
**Glycerol**	5	842	841	842 ±7	0.70 ±0.06
	10	848	847	861 ±7	0.57±0.08
	20	859	857	861 ±9	0.53±0.09
	40	876	873	886 ±13	0.38±0.06
	80	886	883	882 ±18	0.45±0.16

Substrate-concentration dependent *E* shifts under aerobic and anaerobic conditions

FE_{CV}-EPR experiments performed under catalytic conditions at different MBA concentrations were repeated under anaerobic conditions to confirm that the potential shift observed under aerobic conditions was not the result of the presence of atmospheric O_2. The potentials deduced from Nernst fitting the double integrals of the EPR spectra at 1, 5, 10 and 20 mM MBA were plotted against the concentration of substrate (Figure 12). An increase in the potentials obtained from the forward and reverse data sets ($E_{1/2\ forward}$ and $E_{1/2\ reverse}$) as well as their average (E_m) was also observed when increasing the substrate concentration under anaerobic conditions (Figure 12b). The potential shift $E_{m[20\ mM\ MBA]} - E_{m[0mM\ MBA]}$ is +40 mV under both anaerobic and aerobic conditions (Figure 12a). The almost ideal sigmoidal shape of the catalytic CV allows deduction of the catalytic potential, $E_{catalytic(CV)}$ = +881 mV (+879 mV after ohmic drop correction) from the electrochemical data alone, but that FE-EPR provides the first *direct* access to the redox potential of the catalyst during turnover, $E_{catalytic(FE-EPR)}$ = +881 mV, an average of the forward and reverse potentials obtained from fitting EPR double integrals to the Nernst equation (Figure 4c). This is in perfect agreement with the catalytic potential deduced from the CV, $E_{catalytic(CV)}$ (Table 2). The +50 mV shift in potential in the presence of substrate shows that the ratio $STEMPO^{\bullet}$ to $STEMPO^{+}$ has increased during catalysis.

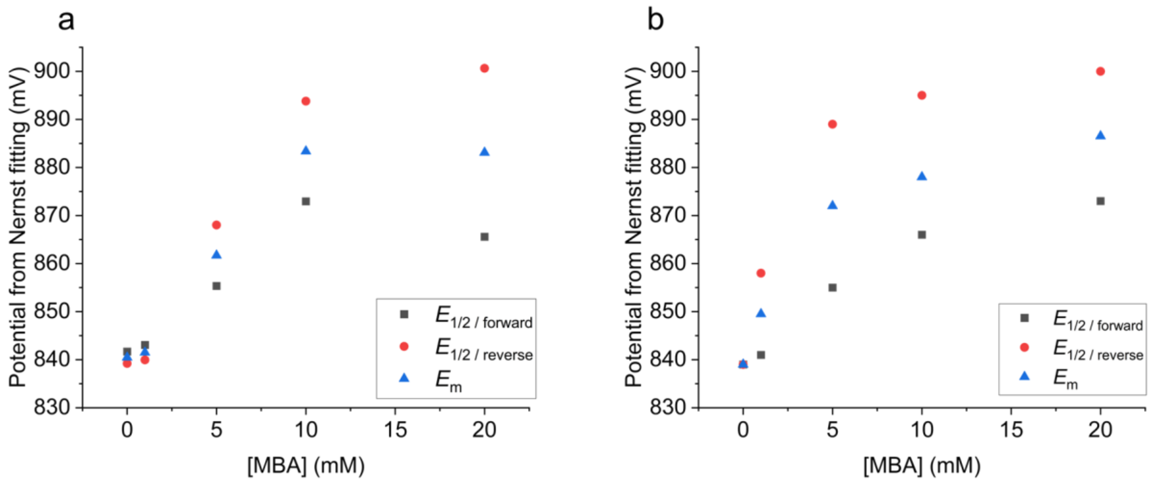

Figure 12. Comparison of the reduction potentials of $STEMPO^{\bullet}/STEMPO^{+}$ deduced from Nernst fitting the FE_{CV}-EPR data performed under: a, aerobic and b, anaerobic conditions in the presence of the substrate MBA (pH 8.0). The FE_{CV}-EPR data under aerobic conditions is shown in Figure 11a. The corresponding primary data under anaerobic conditions was very similar and is not shown.

6. Supporting data for FE_{amp}-EPR experiments

Non-catalytic experiments under anaerobic conditions and at different pH values

FE_{amp}-EPR experiments were performed under anaerobic non-catalytic conditions (Figure 13 left panel), and under aerobic non-catalytic conditions at different pH values (Figure 13 right panel).

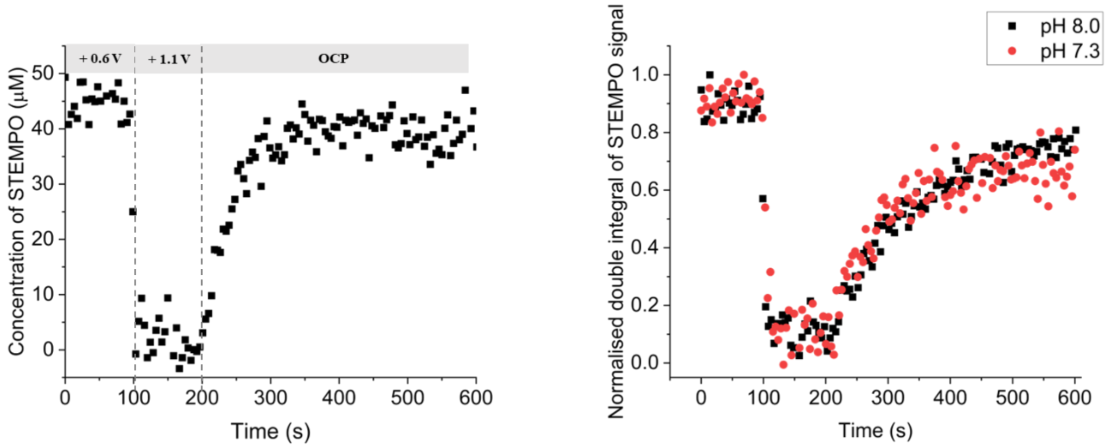

Figure 13. Non-catalytic FE_{amp}-EPR experiment performed under *in situ* N_2 purging at pH 8.0 (left) and under atmospheric O_2 at different pHs (right). EPR spectra taken as a function of time while the potential was held at +0.6 V for 100 s, then stepped to 1.1 V for 100 s, followed by an open circuit potential (OCP) for 400 s. The calibration curve (Fig.10) was used to calculate the concentration of STEMPO from the double integral of EPR signals as shown in panel a, while normalized double integrals of STEMPO signals were considered in the comparison of the regeneration profile of STEMPO at different pHs in the right panel.

During catalysis with MBA under aerobic conditions (pH 8.0)

The regeneration profile of the nitroxide radical at OCP under aerobic conditions and at different concentrations of MBA is shown in Figure 14. The regeneration rate of the EPR-active species increases with increasing substrate concentration. A possible source of electrons enabling the regeneration of STEMPO$^{\bullet}$ from STEMPO^{+} in the absence of substrate is the ITO electrode acting as a capacitor that discharges at OCP[12]. This observation highlights the interest of FE$_{amp}$-EPR in characterising the electronic properties of ITO electrodes. It does not affect the mechanistic investigations of alcohol oxidation by surface-bound oxoammonium.

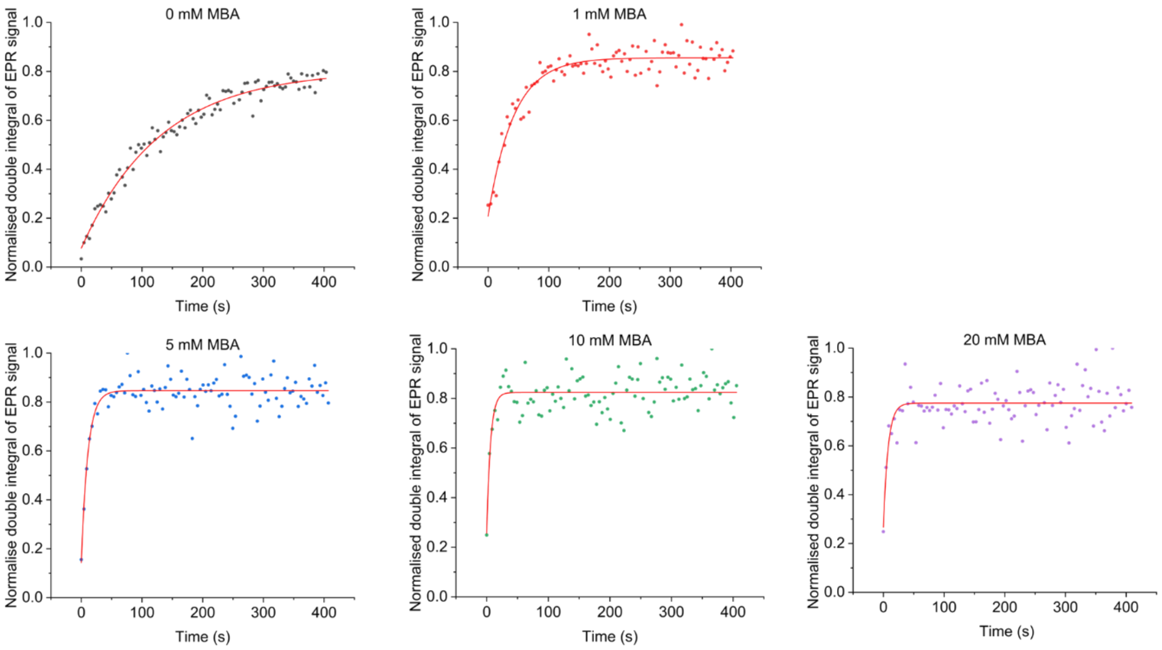

Figure 14. FE$_{amp}$-EPR experiments under aerobic conditions with different MBA concentrations (pH 8.0). Regeneration of STEMPO$^{\bullet}$ at open circuit potential (OCP) after application of high potential step (+1.1 V vs SHE) for 100 s and under different concentrations of MBA: 0 mM, 1 mM, 5 mM, 10 mM and 20 mM. Double integrals of EPR spectra at OCP were normalized to the initial double integral at 0.6 V. A 1st order exponential fit was applied to all data (red lines).

During catalysis with MBA as substrate under anaerobic conditions

As for the result obtained under aerobic conditions, the rate of regeneration of the nitroxide radical under anaerobic conditions at OCP with increasing MBA concentration fits the Michaelis-Menten model (Figure 15).

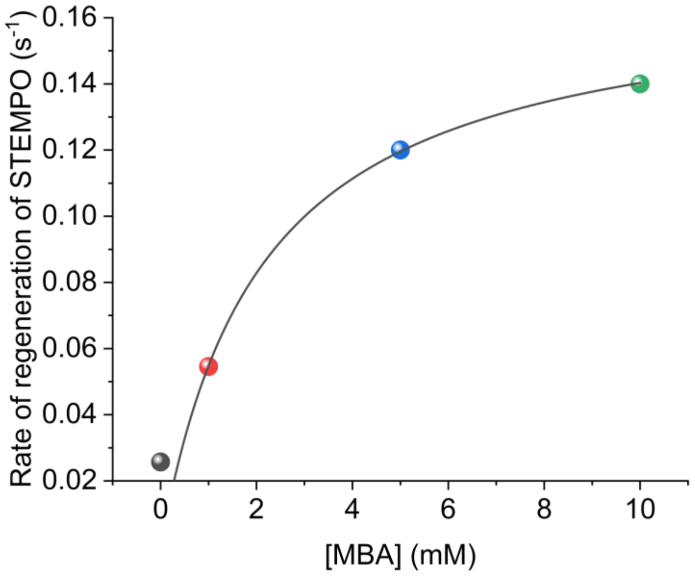

Figure 15. STEMPO• regeneration rates (s^{-1}) as a function of MBA concentrations under anaerobic conditions (pH 8.0). See Figure 5b for comparative data under aerobic conditions. The solid line shows the fit using the Michaelis-Menten model, with K_M = 2.1 mM and k_{cat} = 0.17 s^{-1}.

During catalysis with glycerol under aerobic conditions

Similarly, to FE_{amp}-EPR with MBA, the radical regeneration profile at OCP and different glycerol concentrations (under aerobic conditions) is shown in Figure 16. As for MBA, the regeneration rate of the EPR-active species increased with increasing substrate concentration.

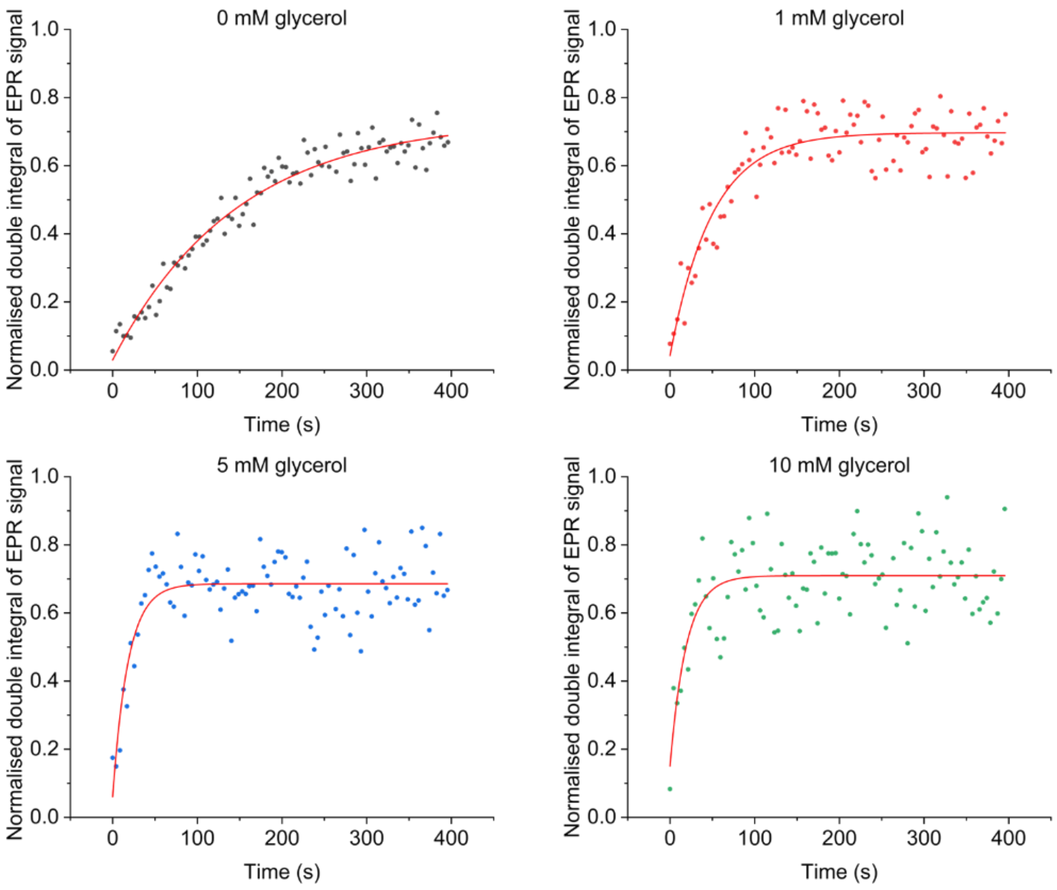

Figure 16. FE_{amp}-EPR experiments under aerobic conditions with different glycerol concentrations (pH 8.0). Regeneration of STEMPO• at open circuit potential (OCP) after application of high potential step (+1.1 V vs SHE) for 100 s and under different concentrations of glycerol: 1 mM, 5 mM, 10 mM and 20 mM. Double integrals of EPR spectra at OCP were normalized to the initial double integral at 0.6 V. A 1st order exponential fit was applied to all data (red lines).

FE_{amp}-EPR experiments with glycerol (Extended data Fig. 8) exhibited similar behaviour to those with MBA (Figure 5a). The radical regenerate rate at OCP increased with increasing glycerol concentration and fits Michaelis-Menten kinetics (Extended data Fig. 8b).

During catalysis with MBA as substrate under aerobic conditions (pH 7.3)

The regeneration profile of the nitroxide radical at OCP under aerobic conditions at pH 7.3 and at different concentrations of MBA is shown in Figure 17. Similar to pH 8.0 (Figure 14), the regeneration rate of the EPR-active species increases with increasing substrate concentration.

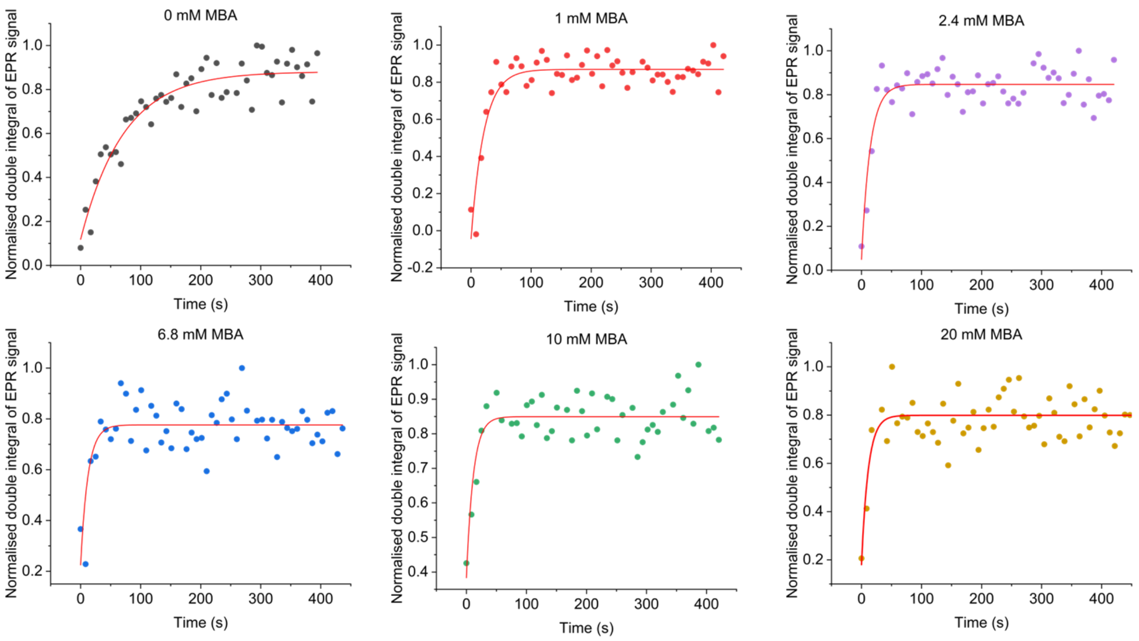

Figure 17. FE_{amp}-EPR experiments under aerobic conditions with different MBA concentrations (pH 7.3). Regeneration of STEMPO$^{\bullet}$ at open circuit potential (OCP) after application of high potential step (+1.1 V vs SHE) for 100 s and under different concentrations of MBA: 0 mM, 1 mM, 2.4 mM, 6.8 mM, 10 mM and 20 mM. Double integrals of EPR spectra at OCP were normalized to the initial double integral at 0.6 V. A 1st order exponential fit was applied to all data (red lines).

Reproducibility

To exemplify reproducibility, an FE_{amp}-EPR experiment with and without substrate is shown in Figure 18. The rate of regeneration of STEMPO after addition of glycerol is perfectly reproducible after cell washing and so is the STEMPO regeneration rate in absence of substrate.

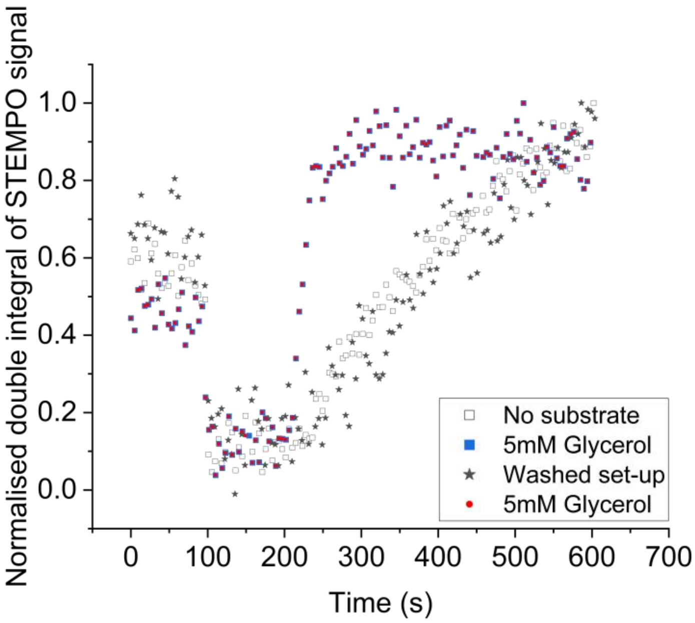

Figure 18. Reproducibility of the FE_{amp}-EPR experiment performed with and without glycerol (pH 8.0). The EPR signal was recorded while holding the potential at 0.8 V vs SHE for 100 s, then the potential was stepped to 1.1 V for 100 s, followed by open circuit potential for 400 s. After recording the data with no substrate (open data points) and 5 mM glycerol (blue data), the FE-EPR cell and working electrodes were rinsed with buffer and the experiment was repeated (dark grey and red data), producing the same results as prior to washing.

Summary of thermodynamic and kinetic parameters

The thermodynamic and kinetic parameters of the oxidation reactions of primary (MBA) and secondary (glycerol) alcohols by surface bound STEMPO as deduced from the FE-EPR experiments are given in Table 3.

Table 3. Thermodynamic and kinetic parameters as deduced from FE-EPR experiments for the reaction of MBA and glycerol by surface-bound STEMPO.

**Substrate**	i_{cat}* CV µA	$E_{cat}/2$* CV mV vs SHE	$E_{cat}/2$* FE_{CV}-EPR mV vs SHE	K_M FE_{amp}-EPR mM	k_{cat} FE_{amp}-EPR s^{-1}
**MBA (pH 8.0)**	89	881	881	6.8 ± 1.3	0.22
**Glycerol (pH 8.0)**	66	859	861	1.8 ± 0.9	0.06

*****Values are deduced from the measurements performed with 20 mM substrate.

7. Michaelis-Menten kinetics in context with the general saturation behaviour model

Our experimental FE_{amp}-EPR data, i.e., the rate of regeneration of radical (y-axis) plotted against the concentration of the substrate (x-axis) (Figure 5, Extended Data Figures 8 and 9), show that each successive incremental decrease (dx) in the concentration of the substrate is less effective at increasing dy. This feature is essential in the description of a saturation behaviour, leading to a hyperbolic distribution of data points that can be fitted using a general second order differential equation as described by Kepner[13]. The Michaelis-Menten model (MM) is one of numerous saturation phenomena that derives from the same general saturation behaviour. It usually describes an enzymatic reaction where a substrate (A) is converted to a product (P) by the catalyst which is the enzyme. The necessary and sufficient condition for this analysis is the experimental data plot the variation over time of P, dP/dt, versus A. The reaction we describe matches this description where the substrate (alcohol) is converted to a product (aldehyde) by the catalyst oxoammonium. The generation of the radical observed by EPR is accelerated in the presence of alcohol during catalysis (Fig. 5) and this is indicative of the catalytic reaction being performed and thus product generation. In other words, the radical state of the catalyst regenerated during the catalysis accompanies the oxidation of alcohol to aldehyde. Therefore, our y axis represents the rate of regeneration of radical during catalysis rather than the direct rate of generation of the product (aldehyde). Supplementary Figure 19 draws parallels between the general saturation behaviour model, MM kinetics and our application of MM, while defining the parameters essential for our applications.

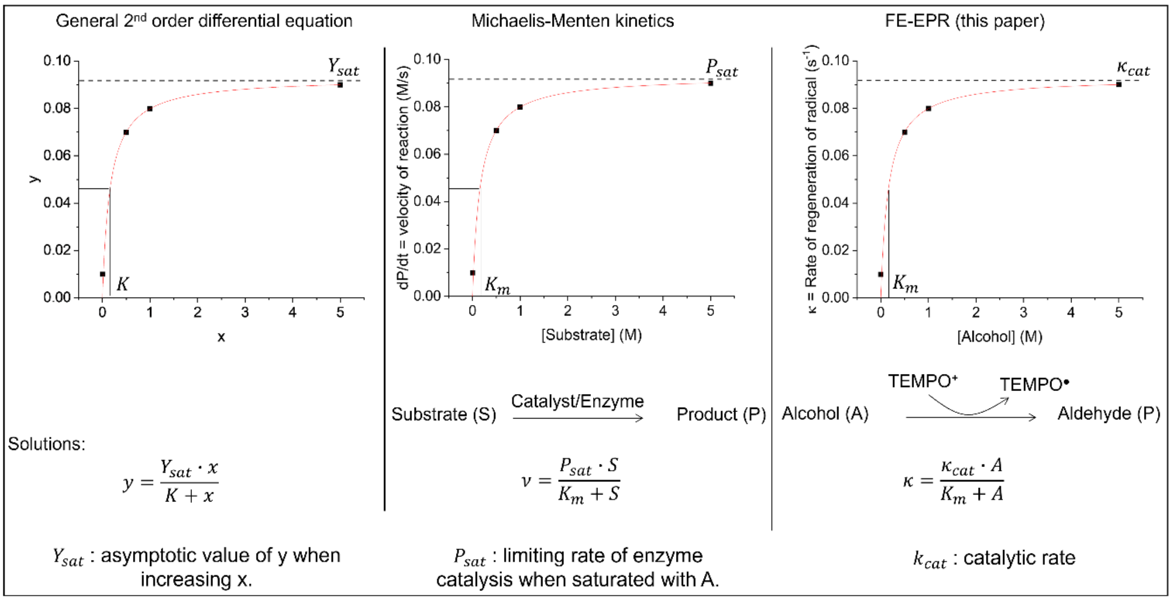

Figure 19. Parallels between the general saturation behaviour model, Michaelis-Menten kinetics and our application of Michaelis-Menten kinetics in this paper.

8. Product detection

To investigate the product of this selective catalytic process, an extended chronoamperometry-OCP measurement was conducted at pH 8.0 with 20 mM MBA as the substrate. 1 mL aliquots were collected prior to and at the end of the catalysis occurring at +1.1 V (Figure 20a). 4-methylbenzaldehyde (MBAd) was determined to be the sole product by high performance liquid chromatography (HPLC) and its amount was quantified by referring to the standard calibration curve (Figure 21). The Faraday efficiency of the reaction was determined as described in ref [1] and found to be 96%.

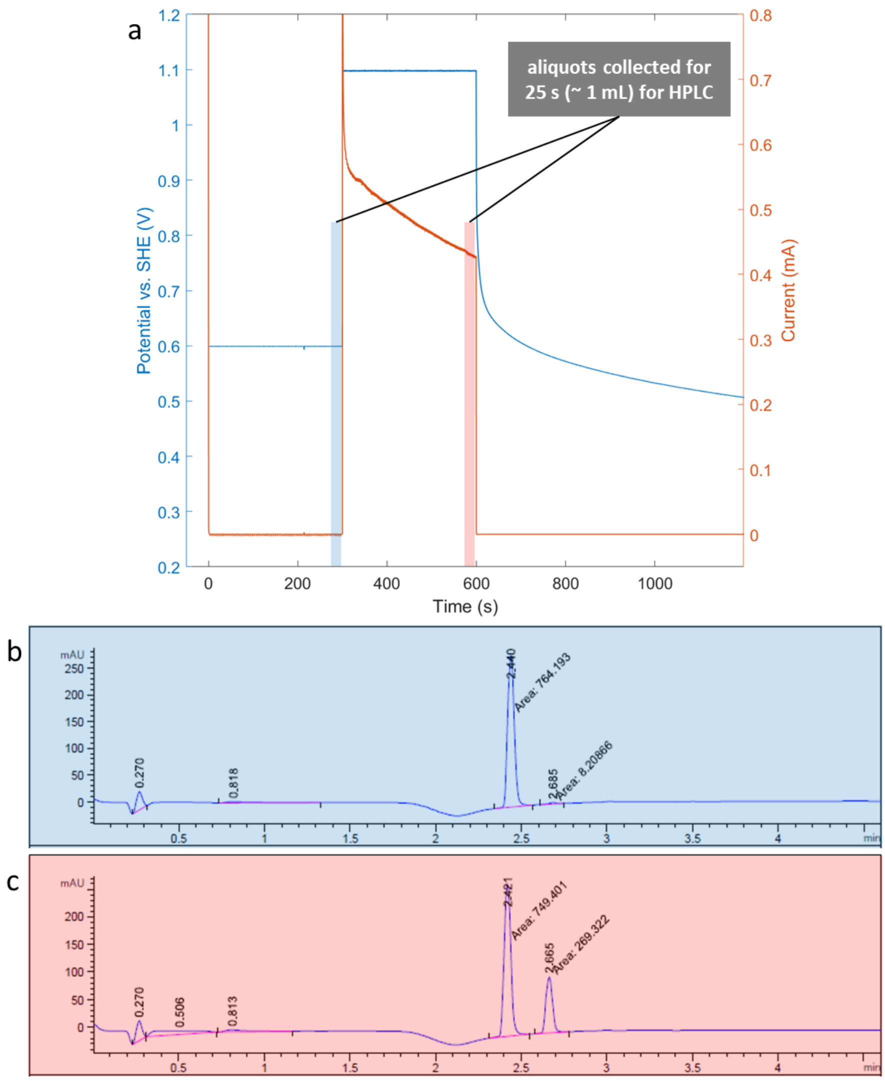

Figure 20. Production detection. a, Chronoamperometry-OCP measurements of STEMPO|mesoITO in the presence of 20 mM MBA (pH 8.0). The potential was held at +0.4 V vs. Ag|AgCl (3M KCl) for 300s where only the EPR-active species (STEMPO•) is present, and then stepped to +0.8 V vs. Ag|AgCl (3M KCl) for 300 s where EPR-silent and catalytically active species ($STEMPO^+$) is generated. After chronoamperometry, the potential was monitored with an open circuit potential measurement for 600s. Conditions: 500 mM carbonate buffer with pH adjusted to 8.0, flow rate ≈ 2 mL /min. **b,** and **c,** HPLC chromatograms (254 nm traces), corresponding to chronoamperometry-OCP measurements with the presence of 20 mM MBA, with a corresponding to the aliquot collected at end of the chronoamperometry at +0.4 V, prior to catalysis occurring at +0.8 V and b corresponding to the aliquot collected at end of the chronoamperometry at +0.8 V, after the catalysis.

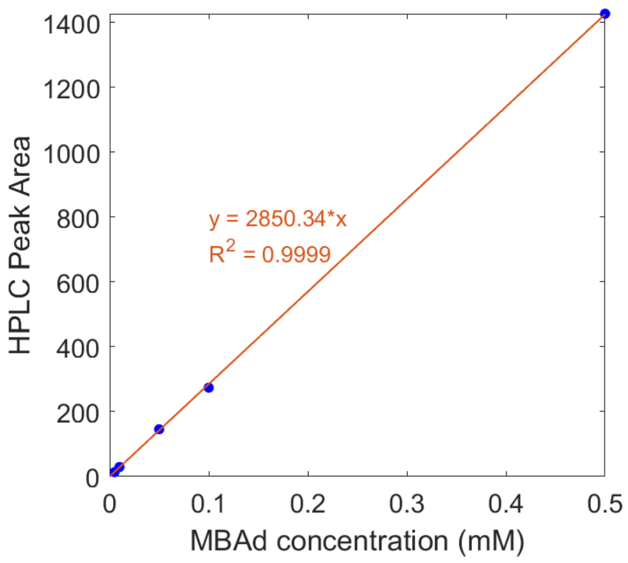

Figure 21. HPLC calibration curve for 4-methylbenzaldehyde (MBAd) detected at 254 nm.

9. Electrochemical analysis

Multiphysics Model Overview

In COMSOL Multiphysics 6.1 a 3D model consisting of the electroanalysis and laminar flow modules were used to describe the solution (electro)chemical reactions and convection, respectively. This model solves ordinary differential equations corresponding to well-defined analytical expressions of mass transport, (electro)chemical reaction rates and equilibria. In-keeping with the observed low cell resistances and minimal ohmic drops, it was assumed that no potential gradients are present (therefore the applied potential is equal to the electrode potential), drastically reducing the model degrees of freedom and computational expense. A time-dependent model was developed where the electrode potential was swept linearly with time to simulate a CV (voltammogram parameters defined in Table 4) and faithfully represent experimental conditions. The model geometry was defined as shown in Figure 22 to represent the experimental cell geometry using dimensions tabulated in Table 5.

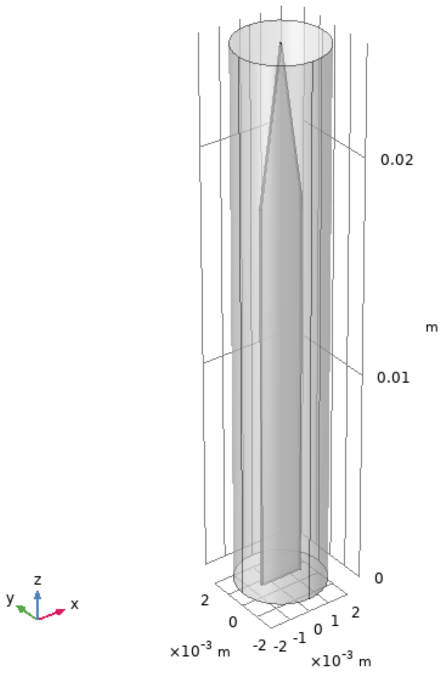

Figure 22 Geometry of 3D COMSOL model showing the solution domain, with interior walls relating to the electrode surface and exterior walls related to the EPR tube.

Electrolyte

Laminar flow

A laminar flow model was constructed to describe the electrolyte flow within the cell. This solves the Navier-Stokes equations (Eq S1–5) for a single-phase flow:

$$\frac{\partial \rho}{\partial t} + \nabla.(\rho \boldsymbol{u}) = 0 \text{ (Eq. S1)}$$

$$p\frac{\partial \boldsymbol{u}}{\partial t} + \rho(\boldsymbol{u}.\boldsymbol{\nabla})\boldsymbol{u} = \nabla.[-p\boldsymbol{I} + \tau] + \mathbf{F} \text{ (Eq. S2)}$$

$$\rho C_p(\frac{\partial T}{\partial t} + (\boldsymbol{u}.\nabla)T) = -(\nabla.\boldsymbol{q}) + \mathbf{t}:\mathbf{S} - \frac{T}{\rho}\frac{\partial p}{\partial t}\Big|_p(\frac{\partial p}{\partial t} + (\boldsymbol{u}.\boldsymbol{\nabla})p) + Q \text{ (Eq. S3)}$$

$$S = \frac{1}{2}(\nabla\boldsymbol{u} + (\nabla\boldsymbol{u})^{\boldsymbol{T}}) \text{ (Eq. S4)}$$

$$\boldsymbol{\tau} = \mathbf{2}\mu\mathbf{S} - \frac{\mathbf{2}}{\mathbf{3}}\mu(\nabla.\boldsymbol{u})\mathbf{I} \text{ (Eq. S5)}$$

where ρ is the density (SI unit: kg m^{-3}), **u** is the velocity vector, p is the pressure, τ is the viscous stress tensor, **F** is the volume force vector, C_p is the specific heat capacity at constant pressure, T is the absolute temperature, **q** is the heat flux vector, Q contains the heat sources, S is the strain-rate tensor. μ represents the dynamic viscosity for a Newtonian fluid that has a linear relationship between stress and strain. The : operation denotes a contraction defined by Eq. S6:

$$\boldsymbol{a}:\boldsymbol{b} = \sum_{\boldsymbol{n}}\sum_{\boldsymbol{m}}\boldsymbol{a}_{\boldsymbol{n}\,\boldsymbol{m}}\boldsymbol{b}_{\boldsymbol{n}\,\boldsymbol{m}} \text{(Eq. S6)}$$

Flow is considered to be incompressible (ρ is constant), a valid assumption for liquids under normal conditions. As such the continuity equation S7 and momentum equation S8 can be written (derived from Eq. S1–2):

$$\rho\nabla.\boldsymbol{u} = \mathbf{0} \text{ (Eq. S7)}$$

$$p\frac{\partial\boldsymbol{u}}{\partial t} + \rho(\boldsymbol{u}.\boldsymbol{\nabla})\boldsymbol{u} = \nabla.[-p\mathbf{I} + \mu(\nabla\mathbf{u} + (\nabla\mathbf{u})^T)] + \mathbf{F} \text{ (Eq. S8)}$$

Transport of diluted species (electroanalysis)

The transport of species obeys the Nernst-Planck equation for the flux of species i (Eq. S9):

$$\boldsymbol{N}_i = -D_i\nabla c_i - z_i u_{m,i} F c_i \nabla\Phi_l + c_i\boldsymbol{u} \text{ (Eq. S9),}$$

which simplifies to Equation S10 under no electric field (as at high electrolyte concentrations):

$$\boldsymbol{N}_i = -D_i\nabla c_i + c_i\boldsymbol{u} \text{ (Eq. S10).}$$

A mass balance is also applied to each chemical species (Equation S11):

$$\frac{\partial c_i}{\partial t} + \nabla.\boldsymbol{N}_i = R_{i,tot} \text{(Eq. S11).}$$

Wall boundary conditions

Solid Walls of domains representing the electrode surface and the walls of the EPR tube were represented with a no slip boundary condition Eq. S12:

$$\boldsymbol{u} = 0 \text{ (Eq. S12)}$$

A no flux boundary condition is also applied (Eq. S13)

$$-\boldsymbol{n}(\boldsymbol{J}_{\boldsymbol{i}} + \boldsymbol{u}c_i) = 0 \text{ (Eq. S13)}$$

These walls are also considered to act as insulation and no current can flow (Eq. S14)

$$-\boldsymbol{n}.\boldsymbol{i}_{\boldsymbol{s}} = 0 \text{ (Eq. S14)}$$

Inlet

A fully developed laminar inflow condition was applied to the inlet to represent the EPR tube above and the electrode. Here a boundary adds a weak form contribution and constraints corresponding to unidirectional flow perpendicular to the boundary. The average inlet velocity was specified according to the flow rate and inlet dimensions (Eq. S15–S16):

$$U_{av} = -\frac{1}{A}\int_{\partial\Omega_{inl}} \boldsymbol{u}.\boldsymbol{n}\, dS \text{ (Eq. S15)}$$

$$A = \int_{\partial\Omega_{inl}} dS \text{ (Eq. S16)}$$

where Ω is the domain on which the inlet resides. This inflow is considered to be time invariant (Eq S17):

$$\boldsymbol{u}.\boldsymbol{t} = 0 \text{ (Eq. S17)}$$

The concentration of species in the inlet solution is fixed by a concentration boundary condition to the inlet concentration value (Eq. S18)

$$c_i = c_{0,i} \text{ (Eq. S18)}$$

Outlet

A fully developed laminar outflow condition was applied to the outlet to represent the EPR tube below the electrode. As for the inlet the flow is considered to be invariant with time (Eq. S17). The average outlet pressure is defined using equations S19–20:

$$p_{av} = \frac{1}{A}\int_{\partial\Omega_{out}} p\, dS \text{ (Eq. S19)}$$

$$A = \int_{\partial\Omega_{out}} dS \text{ (Eq S20)}$$

At the outlet contribution dominates fluid flow and convection is negligible, as such a boundary condition of the form of Eq. S21 can be defined:

$$\boldsymbol{n}.(-D\nabla c) = 0 \text{ (Eq. S21)}$$

Electrode Surface

In addition to the wall conditions regarding solution flow and concentrations, additional conditions were applied to the electrode surface to represent electron transfer and surface chemistry. The oxidation of the surface immobilised STEMPO molecule (Equation S22) was represented by an asymmetric Marcus-Hush-Chidsey description that takes into account intermolecular interactions. The rate of surface reaction (R_{et}) was defined by Equation S23 and the current by equations (S24–S29) This was required to faithfully represent the observed voltammograms in the absence of substrate over all scan rates (Extended Data Fig. 4). Parameters used are tabulated in Table 6.

$$STEMPO^{\bullet} \overset{R_{et}}{\leftrightarrow} STEMPO^{+} + 1e^{-} \text{ (Eq. S22)}$$

$$R_{et} = \frac{I}{nF} \text{ (Eq. S23)}$$

$$I = \Gamma_T k_s . g(\eta_{ap}) e^{-\vartheta_E S \theta_r} \left(\theta_r e^{-\eta_{ap}\vartheta_E G \theta_o} - \theta_o e^{\vartheta_E G \theta_r}\right) \text{(Eq. S24)}$$

$$\theta_r = \frac{\Gamma_r}{\Gamma_T} \text{ (Eq. S25)}$$

$$\theta_o = \frac{\Gamma_o}{\Gamma_T} \text{(Eq. S26)}$$

$$g(\eta_{ap}) = \frac{F(\eta_{ap}, \Lambda)}{F(0,\Lambda)} = \frac{\int_{-\infty}^{\infty}\left(\frac{e^{-\Delta G^{\ddagger}_{red}(\varepsilon,\eta_{ap},\Lambda,\gamma)}}{1+e^{-\varepsilon}}\right)d\varepsilon}{\int_{-\infty}^{\infty}\left(\frac{e^{-\Delta G^{\ddagger}_{red}(0,\eta_{ap},\Lambda,\gamma)}}{1+e^{-\varepsilon}}\right)d\varepsilon} \text{(Eq. S27)}$$

$$\Delta G^{\ddagger}_{red}(\varepsilon,\eta,\Lambda,\gamma) = \frac{\Lambda}{4}(1+\frac{\eta+\varepsilon}{\Lambda})^2 + \gamma\left(\frac{\eta+\varepsilon}{4}\right)\left(1-\left(\frac{\eta+\varepsilon}{\Lambda}\right)^2\right) + \gamma^2\frac{\Lambda}{16} \text{(Eq. S28)}$$

$$\eta = \frac{F}{RT}(E - E_0) \text{ (Eq. S29)}$$

where $\vartheta_E S$ and $\vartheta_E G$ are dimensionless constants representing the intermolecular reactions between STEMPO species on the electrode surface, k_s is the electron transfer rate constant, Γ_T is the total surface coverage of STEMPO molecules (mol m^{-2}) , Γ_r is the surface coverage of reduced STEMPO and Γ_o is the surface coverage of oxidised STEMPO, η is the dimensionless overpotential, Λ is the dimensionless reorganization energy, γ is the asymmetry parameter and ε is an integration parameter. All parameters used are listed in Table 6.

In addition to the surface bound faradaic current, a double layer capacitance current was added (Eq. S30):

$$i_{dl} = \left(\partial\frac{(\emptyset_{s,ext} - \emptyset_l)}{\partial t}\right) C_{dl} \text{ (Eq. S30)}$$

where $\emptyset_{s,ext}$ is the electrode potential and $\emptyset_l$ is the electrolyte potential, with the term in brackets equivalent to the scan rate (change in potential with time) and C_{dl} being the double layer capacitance (F m^{-2}).

In addition to faradaic reactions on the electrode surface (Eq. 1 & 6), non-faradaic surface chemical reactions were included related to STEMPO-adduct formation (Eq. 2), where K_{eq} is the equilibrium constant, and alcohol oxidation reaction (Eq. 4) to form STEMPOH and an aldehyde. A non-faradaic comproportionation reaction (Eq. 5) was included as a potential route for $TEMPO^{\bullet}$ regeneration from TEMPOH, this was substituted for a faradaic PCET regeneration route (Eq. 6) where indicated. The pH dependent formation of an oxoammonium hydroxide adduct (Eq. 3) was also included.

Overall rates of surface species consumption and generation were represented by Eq. S31:

$$\frac{\Gamma}{\sigma_k}\frac{d\theta_{s,k}}{dt} = \sum_m R_{et,k,m} + \sum_n R_{nf,k,n} \text{ (Eq. S31)}$$

Supplementary Figures for Multiphysics modelling

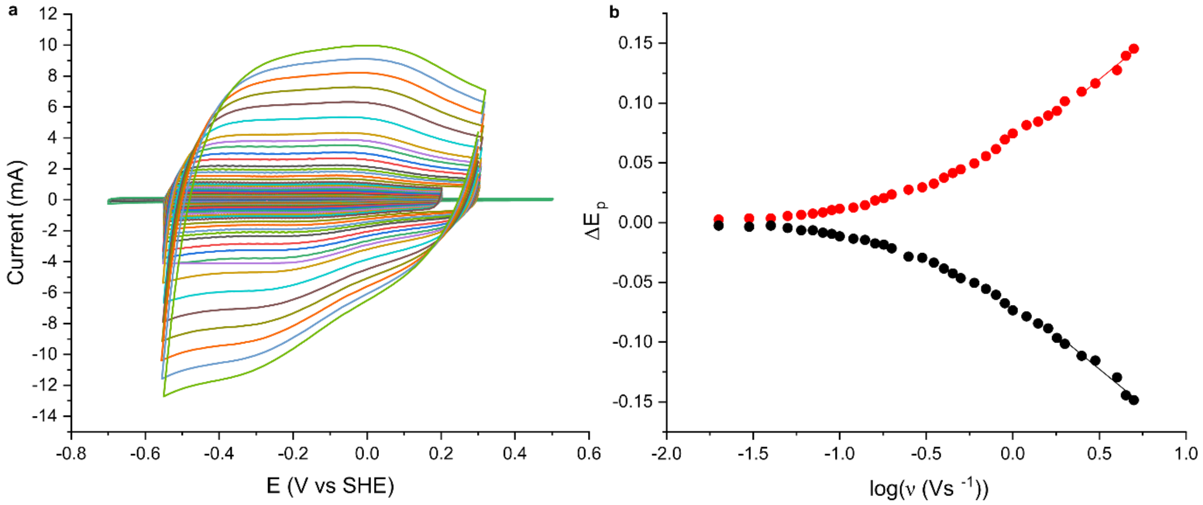

Figure 23. Non-turnover signals for STEMPO related to the STEMPOH/STEMPO$^{\bullet}$ redox couple. **a,** Cyclic voltammograms of STEMPOH/STEMPO$^{\bullet}$ redox couple on a mesoITO electrode over a range of scan rates (5 – 5000 mV) at pH 8.0. **b**, Experimental peak potentials for the anodic (red) and cathodic (black) branch. Lines represent linear fits to the region of experimental data where $\Delta E_p > 200$ mV for experimental determination of $k_s = 5.7\ s^{-1}$ and $\alpha = 0.51$[14].

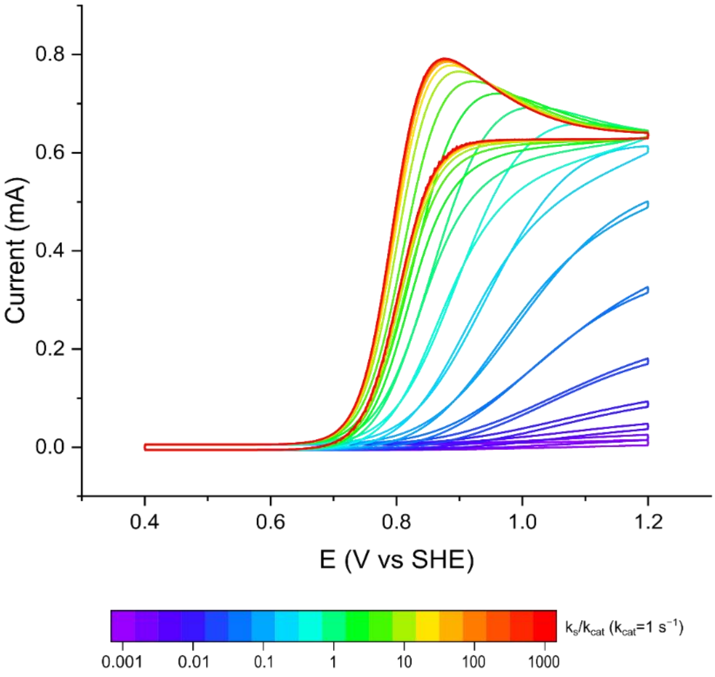

Figure 24. Effect of varying k_S on the catalytic response. CVs at a range of k_s from 10^{-3}–10^{3} s^{-1} with k_{cat} fixed at 1 s^{-1}.

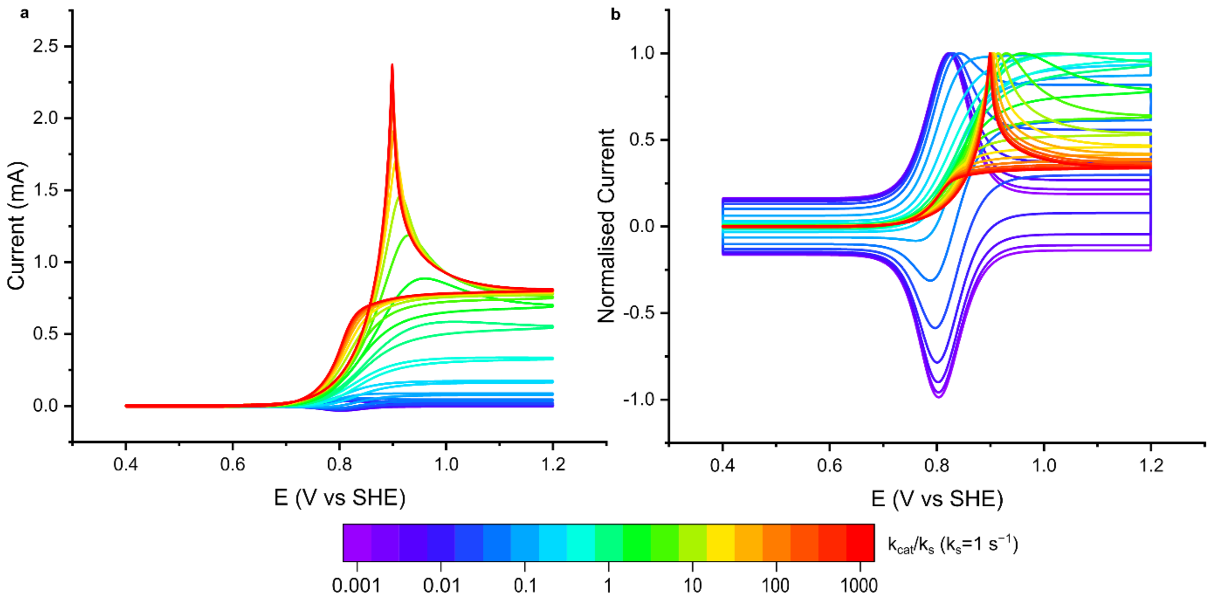

Figure 25. Effect of varying k_{cat} on the catalytic response. a, CVs at a range of k_{cat} from 10^{-3}–10^{3} s^{-1} with k_s fixed at 1 s^{-1}. **b,** Normalised CVs at a range of k_{cat} from 10^{-3}–10^{3} s^{-1} with k_s fixed at 1 s^{-1}.

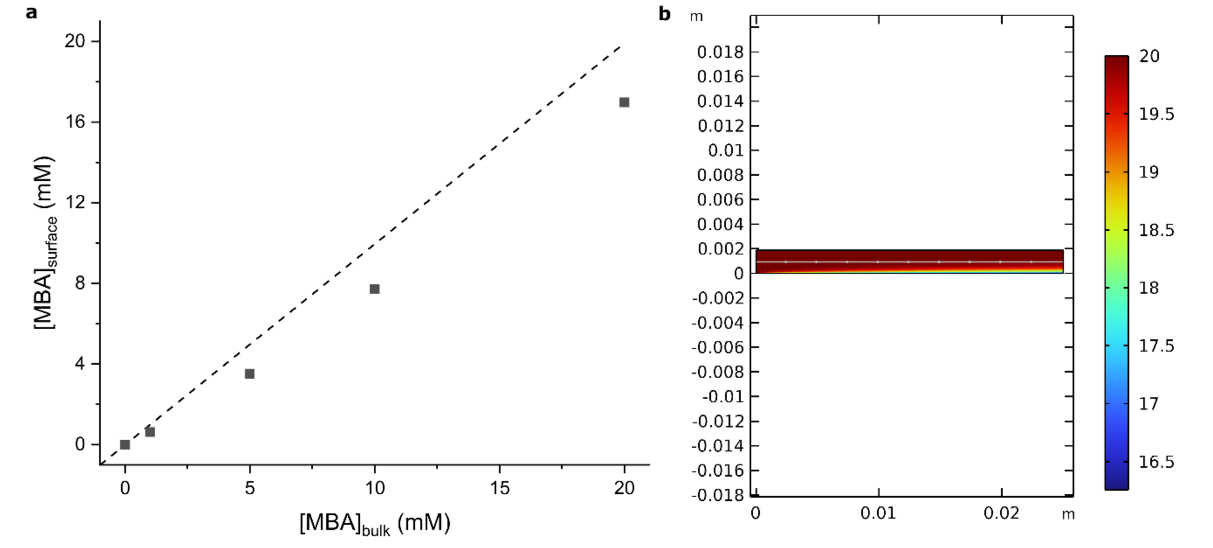

Figure 26. Surface Concentrations of MBA from Multiphysics Modelling. **a**, Dependence of average surface concentrations of MBA on substrate concentration, demonstrating limited substrate depletion under the mass transport conditions used. **b**, Exemplar 2D concentration profile of MBA within the cell (t = 160 s, E_{app} = +1.2 V vs SHE, $[MBA]_{bulk}$ = 20 mM), where the inlet is located at the boundary parallel to the y axis at x = 0 and the outlet is the boundary parallel to the x axis at x = 0.025 m. The electrode surface is located parallel to the x axis at y = 0. Due to solution flow parallel to the electrode surface the concentration profile in y is not homogenous, however the effect on surface concentration is limited.

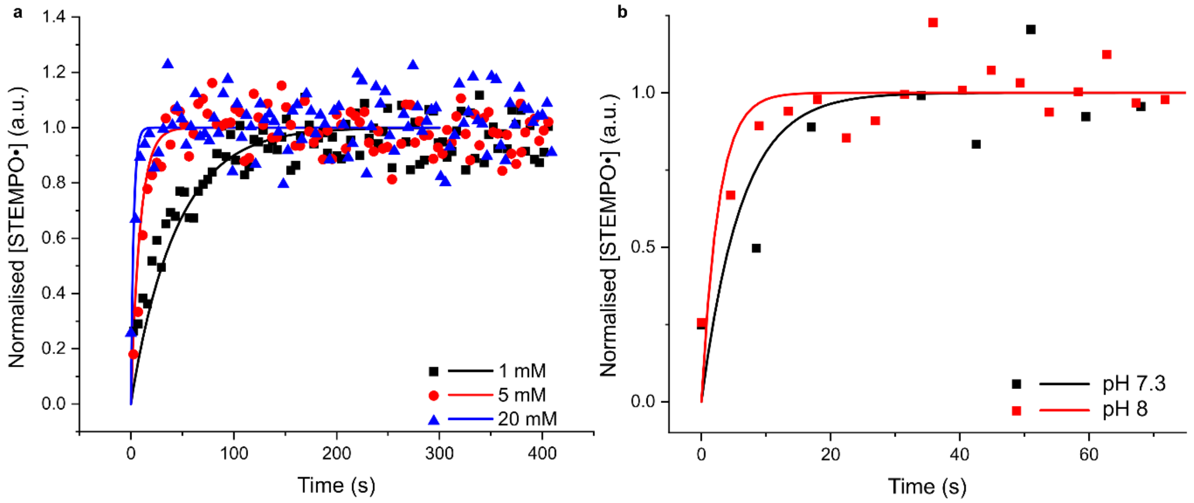

Figure 27. Experimental and simulated FE-EPR experiments at OCP. a, EPR (points) and simulated (lines) STEMPO$^{\bullet}$ concentrations at pH 8 for 1 mM (black), 5 mM (red) and 20 mM (blue) MBA. **b,** EPR (points) and simulated (lines) STEMPO$^{\bullet}$ concentrations at pH 7.3 (black) and pH 8 (red) for 20 mM MBA.

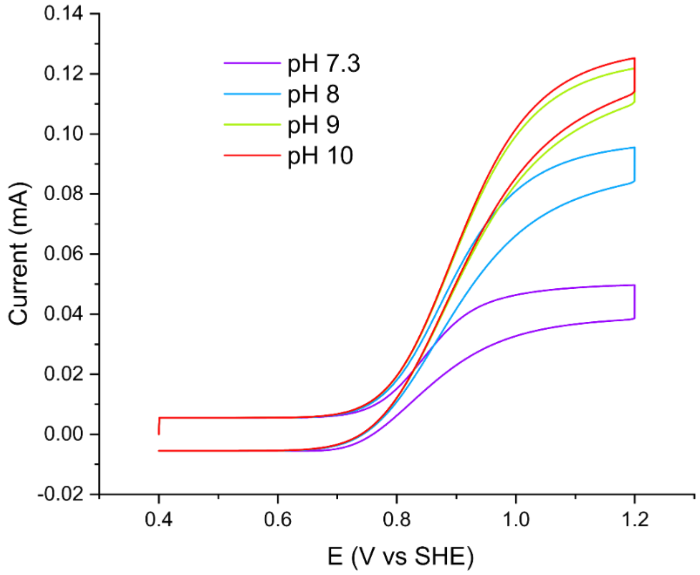

Figure 28. Simulated CVs for alcohol oxidation by STEMPO at a range of pH. CVs showing an increasing current with pH, plateauing at high pH due to the formation of STEMPO-OH adduct (see main paper Eq. 3).

Supplementary Tables for Multiphysics modelling

Table 4. Voltammogram parameters used for multiphysics modelling

Parameter	Value
E_{start}	0.4 V
E_{vertex}	1.2 V
E_{end}	0.4 V
v	5 mV s^{-1}

Table 5. Geometric parameters used for multiphysics modelling

Parameter	Value
Electrode thickness	0.127 mm
Electrode length	25 mm
Electrode taper length	7 mm
Tube internal diameter	2 mm

Table 6. Kinetic parameters used for multiphysics modelling

Parameter	Value
k_s	Experimentally determined (non turnover= 0.77 s^{-1}, FE-EPR= 0.037 s^{-1})
K_m	3.2 mM
$k_{cat,pH\ dependent}$	780 m^3 s^{-1} mol^{-1}
$k_{s,pcet}$	5.7 s^{-1}
$\vartheta_E S$	−0.4
$\vartheta_E G$	0.15
Λ	7
γ	0.15

10. Foot of the wave analysis

Foot of the wave analysis[15] was used to extract the pseudo first order rate constant (2k) for the system. A plot of current divided by the no substrate peak current was plotted against the dimensionless parameter (x) and the slope of the linear regime determined. Following eq. S32 the pseudo first order rate constant was extracted ($2kC_{MBA}$) and plotted against C_{MBA} (Figure 29b to determine the overpotential dependent $TOF^{(2)}$ Figure 29c and $TOF^{(2)}_0$ ($TOF^{(2)}_0$= 3.75×10^{-14} s^{-1}). The overpotential was calculated using the pH dependent formal potential of MBA and MBAd was calculated to be (E^0= –0.026–0.059×pH) using the Gibbs Free energies (ΔG=–nFE^0). This analysis, while extracting suitable constants is not typically applied for Michaelis-Menten style systems and is superseded by the more comprehensive Multiphysics Modelling completed in this work.

$$\frac{1}{1+e^{\frac{F}{RT}(E^0_{PQ}-E)}} \quad (x)$$

$$\frac{i}{i_{p,0}} = \frac{2.24\sqrt{\frac{RT}{Fv}2kC^0_{MBA}}}{1+e^{\frac{F}{RT}(E^0_{PQ}-E)}} \quad \text{(Eq. S32)}$$

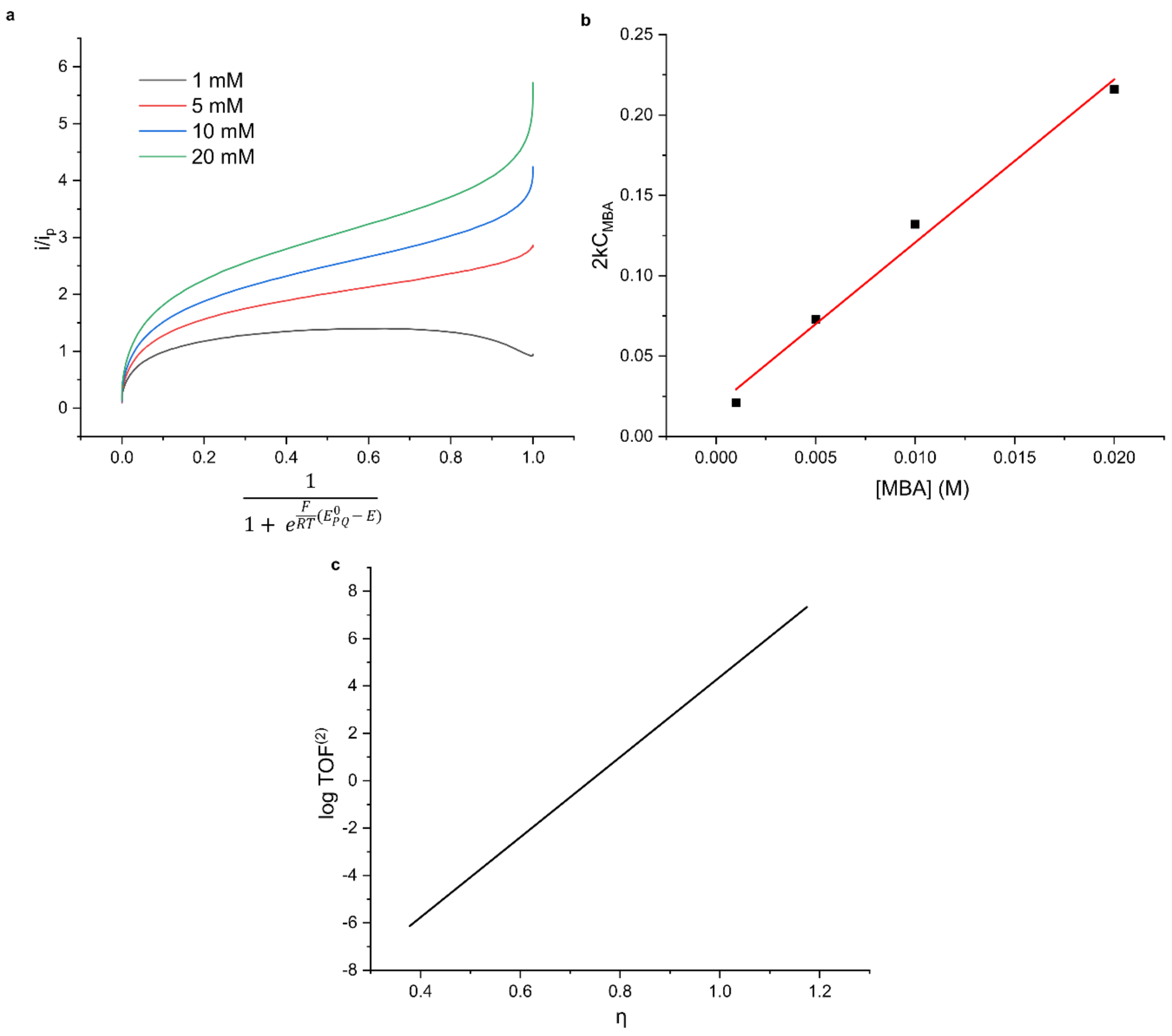

Figure 29. Foot of the wave analysis. a plot of i/i_p against the dimensionless parameter to determine the linear region. **b** plot of $2kC_{MBA}$ vs MBA concentration (C_{MBA}) to determine 2k. **c** log $TOF^{(2)}$ overpotential dependence

11. References

1. Bajada, M. A. *et al.* A Precious-Metal-Free Hybrid Electrolyzer for Alcohol Oxidation Coupled to CO(2) -to-Syngas Conversion. *Angew Chem Int Ed Engl* **59**, 15633–15641 (2020).

2. Laviron, E. General expression of the linear potential sweep voltammogram in the case of diffusionless electrochemical systems. *J Electroanal Chem Interfacial Electrochem* **101**, 19–28 (1979).

3. Semmelhack, M. F., Schmid, C. R. & Cortés, D. A. Mechanism of the oxidation of alcohols by 2,2,6,6-tetramethylpiperidine nitrosonium cation. *Tetrahedron Lett* **27**, 1119–1122 (1986).

4. Golubev, V. A., Rozantsev, E. G. & Neiman, M. B. Some reactions of free iminoxyl radicals with the participation of the unpaired electron. *Bulletin of the Academy of Sciences, USSR Division of Chemical Science* **14**, 1898–1904 (1965).

5. Kishioka, S., Ohsaka, T. & Tokuda, K. Spectroelectrochemical Detection of an Intermediate in the Alcohol Oxidation Process with a Nitroxyl Radical. *Chem Lett* **27**, 343–344 (1998).

6. Bailey, W. F., Bobbitt, J. M. & Wiberg, K. B. Mechanism of the Oxidation of Alcohols by Oxoammonium Cations. *J Org Chem* **72**, 4504–4509 (2007).

7. Nutting, J. E., Rafiee, M. & Stahl, S. S. Tetramethylpiperidine N-Oxyl (TEMPO), Phthalimide N-Oxyl (PINO), and Related N-Oxyl Species: Electrochemical Properties and Their Use in Electrocatalytic Reactions. *Chem Rev* **118**, 4834–4885 (2018).

8. Semmelhack, M. F., Schmid, C. R. & Cortés, D. A. Mechanism of the oxidation of alcohols by 2,2,6,6-tetramethylpiperidine nitrosonium cation. *Tetrahedron Lett* **27**, 1119–1122 (1986).

9. Comminges, C., Barhdadi, R., Doherty, A. P., O'Toole, S. & Troupel, M. Mechanism of 2,2′6,6′-Tetramethylpiperidin- *N* -oxyl-Mediated Oxidation of Alcohols in Ionic Liquids. *J Phys Chem A* **112**, 7848–7855 (2008).

10. Rafiee, M., Miles, K. C. & Stahl, S. S. Electrocatalytic Alcohol Oxidation with TEMPO and Bicyclic Nitroxyl Derivatives: Driving Force Trumps Steric Effects. *J Am Chem Soc* **137**, 14751–14757 (2015).

11. Taitt, B. J., Bender, M. T. & Choi, K. S. Impacts of the regeneration pathways of the oxoammonium cation on electrochemical nitroxyl radical-mediated alcohol oxidation. *ACS Catal* **10**, 265–275 (2020).

12. Bellani, S. *et al.* ITO nanoparticles break optical transparency/high-areal capacitance trade-off for advanced aqueous supercapacitors. *J Mater Chem A Mater* **5**, 25177–25186 (2017).

13. Kepner, G. R. Saturation Behavior: A general relationship described by a simple second-order differential equation. *Theor Biol Med Model* **7**, 1–13 (2010).

14. Laviron, E. General expression of the linear potential sweep voltammogram in the case of diffusionless electrochemical systems. *Journal of Electroanalytical Chemistry* **101**, 19–28 (1979).

15. Costentin, C., Drouet, S., Robert, M. & Savéant, J. M. Turnover numbers, turnover frequencies, and overpotential in molecular catalysis of electrochemical reactions. Cyclic voltammetry and preparative-scale electrolysis. *J Am Chem Soc* **134**, 11235–11242 (2012).
