# Supplementary material for: Operando film-electrochemical EPR spectroscopy tracks radical intermediates in surface-immobilized catalysts
Source: Nat Chem. 2024 Feb 14;16(6):1015–23. doi: 10.1038/s41557-024-01450-y (PMC11636982; doi:10.1038/s41557-024-01450-y)
Supplement: Supplementary file 4 — Unprocessed confocal image. [file 41557_2024_1450_MOESM4_ESM.pdf]

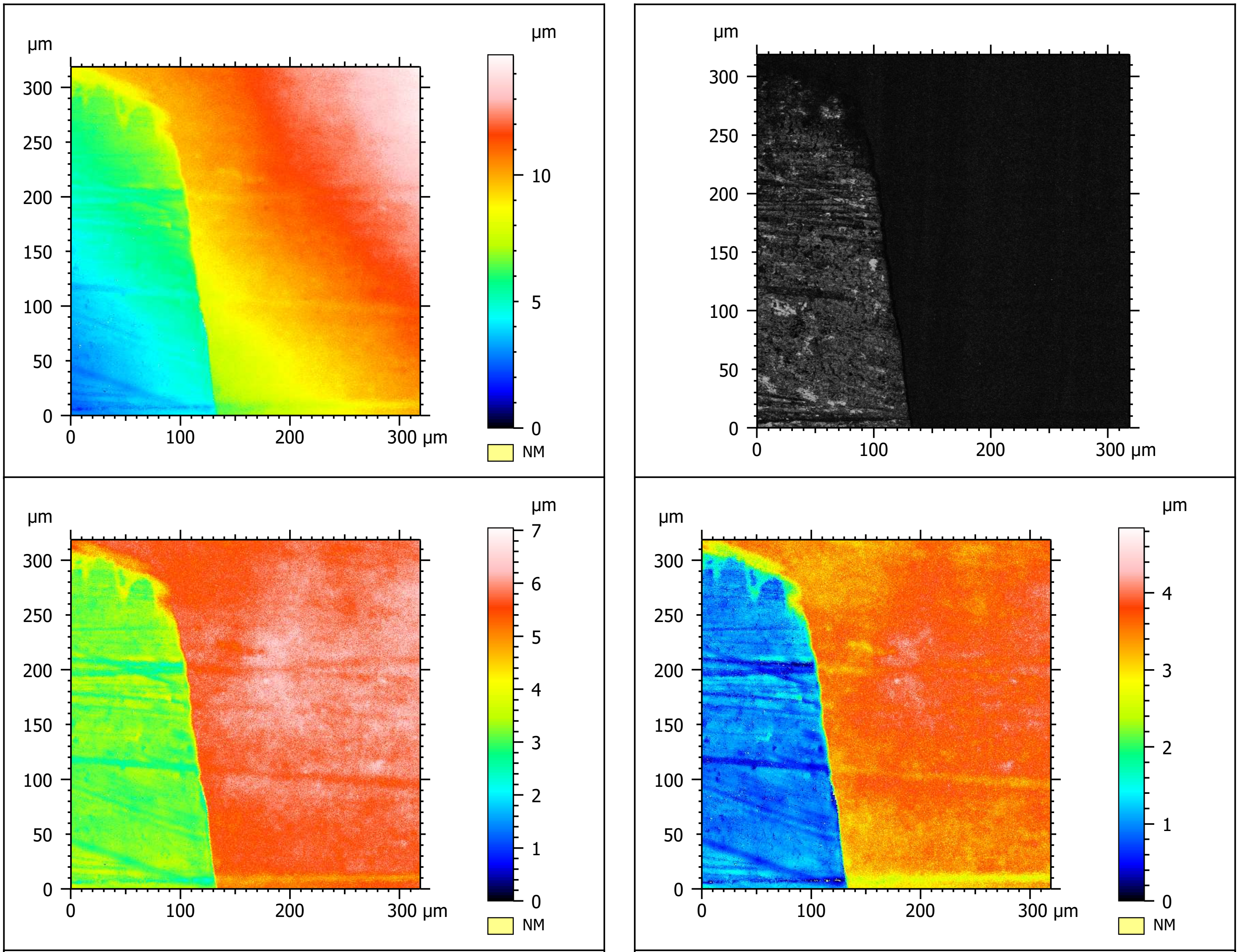

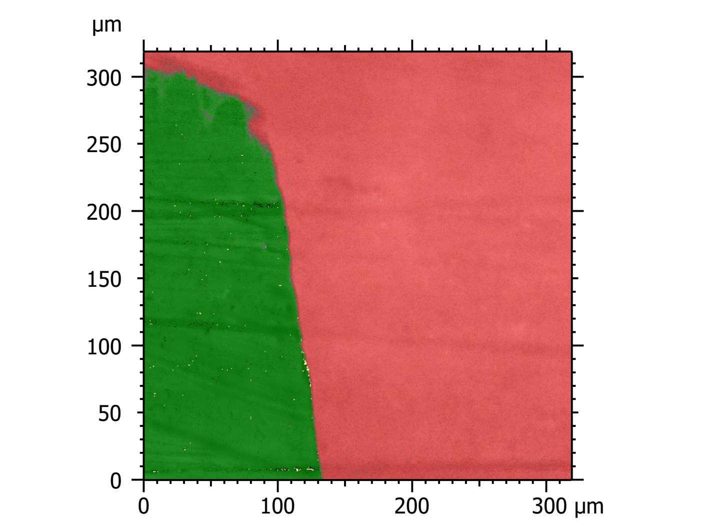

Differential parameters	P2 - P1	Unit	
Zmean(higher) - Zmean(lower)	2.580	µm	
Angle difference	0.1142	°	

Plane parameters	Unit	Plane 1	Plane 2
Zmin	µm	0.1459	2.181
ZMean	µm	0.9752	3.555
Zmax	µm	1.793	4.647

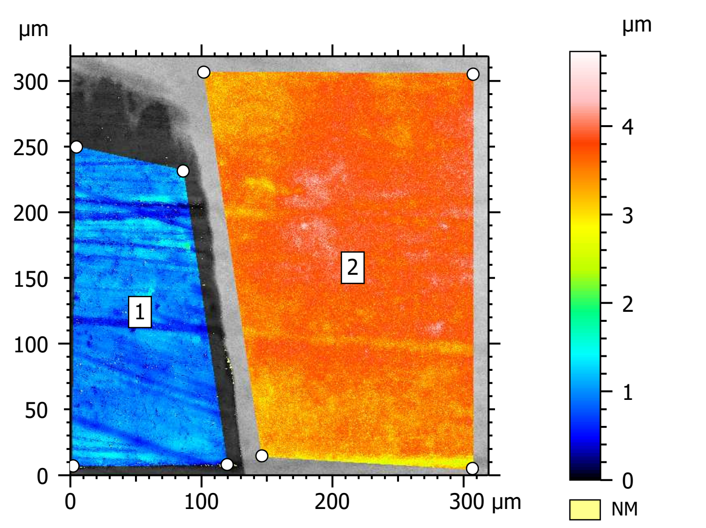

Differential parameters	P2 - P1	Unit	
Zmean(higher) - Zmean(lower)	2.662	µm	
Angle difference	0.09436	°	

Plane parameters	Unit	Plane 1	Plane...
Zmin	µm	0.000	2.051
ZMean	µm	0.9498	3.612
Zmax	µm	4.846	4.757

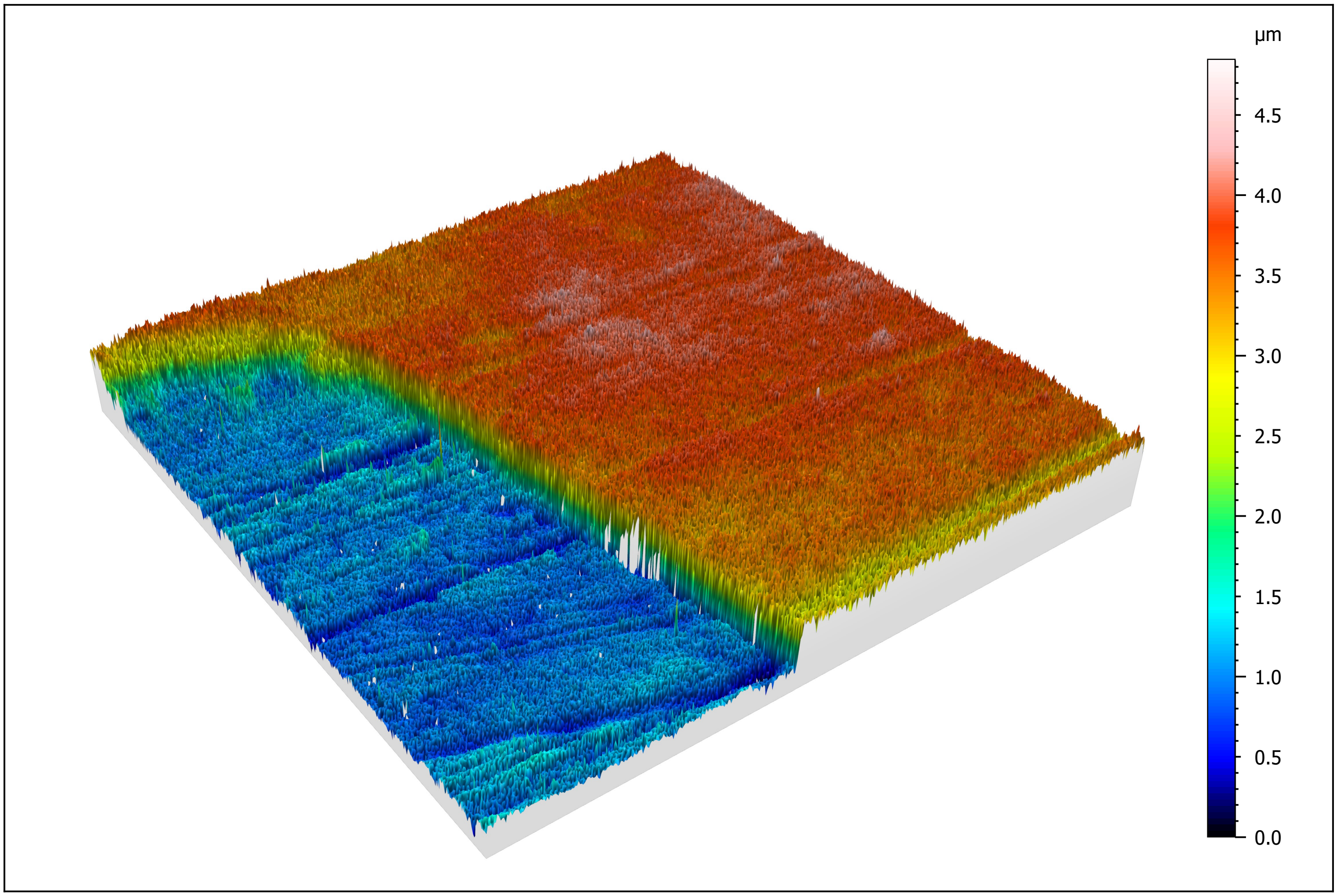
µm
4.5
4.0
3.5
3.0
2.5
2.0
1.5
1.0
0.5
0.0

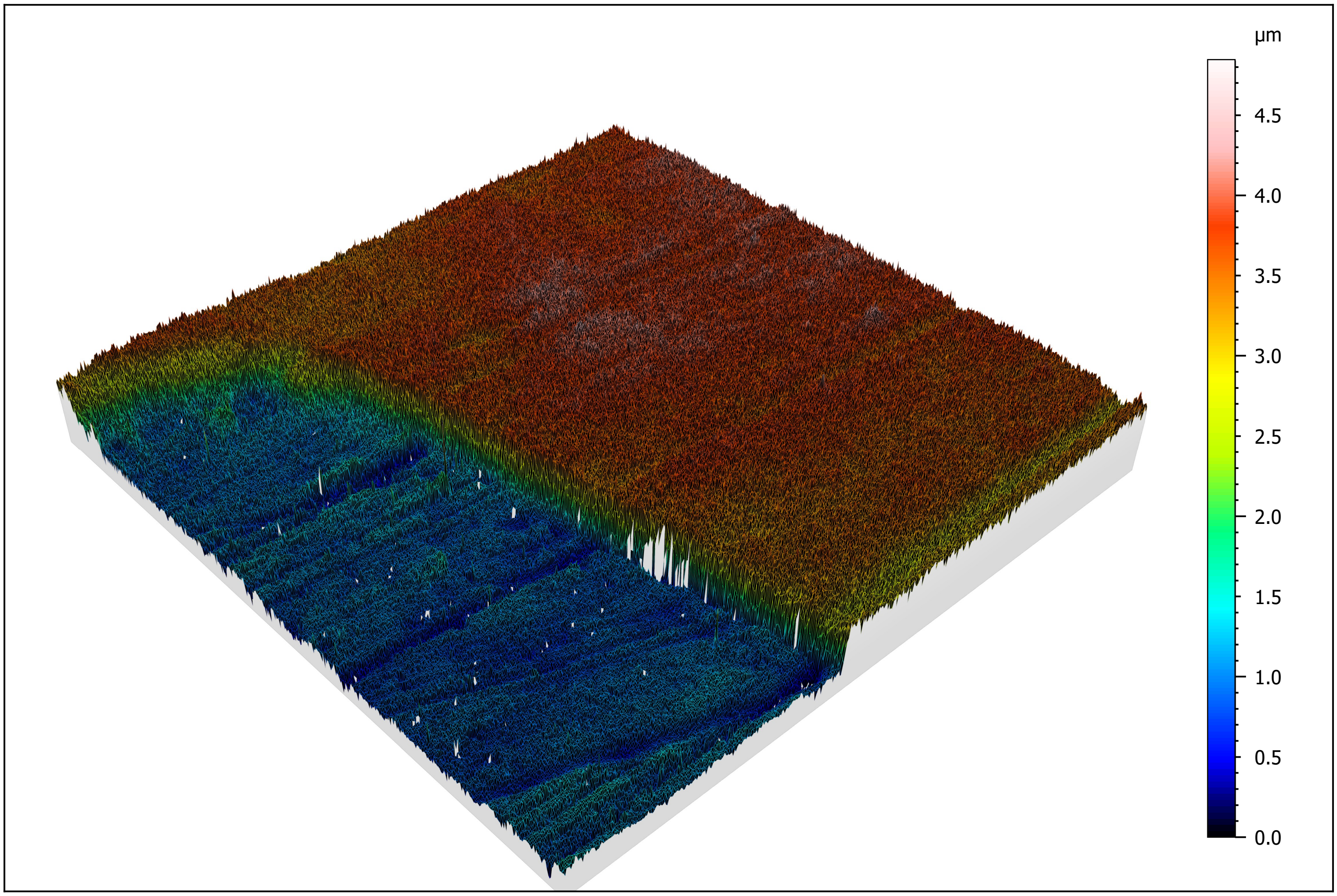
µm
4.5
4.0
3.5
3.0
2.5
2.0
1.5
1.0
0.5
0.0
